# The Cancer Therapy-Related Clonal Hematopoiesis Driver Gene *Ppm1d* Promotes Inflammation and Non-Ischemic Heart Failure in Mice

**DOI:** 10.1161/CIRCRESAHA.121.319314

**Published:** 2021-07-28

**Authors:** Yoshimitsu Yura, Emiri Miura-Yura, Yasufumi Katanasaka, Kyung-Duk Min, Nicholas Chavkin, Ariel H. Polizio, Hayato Ogawa, Keita Horitani, Heather Doviak, Megan A. Evans, Miho Sano, Ying Wang, Katharina Boroviak, George Philippos, Ana Filipa Domingues, George Vassiliou, Soichi Sano, Kenneth Walsh

**Affiliations:** 1 Hematovascular Biology Center, Robert M. Berne Cardiovascular Research Center, University of Virginia School of Medicine, Charlottesville, VA (Y.Y., E.M.-Y., K.-D.M., N.C., A.H.P., H.O., K.H., H.D., M.A.E., M.S., Y.W., S.S., K.W.).; 2 Now with Division of Molecular Medicine, Graduate School of Pharmaceutical Sciences, University of Shizuoka, Yada, Japan (Y.K.).; 3 Department of Cardiology, Xinqiao Hospital, Army Medical University, Chongqing, China (Y.W.).; 4 Wellcome Sanger Institute, Wellcome Genome Campus, Cambridge, United Kingdom (K.B., G.P., G.V., A.F.D.).; 5 Now with Interfaculty Institute of Cell Biology, Eberhard Karls University Tübingen, Germany (G.P.).; 6 Wellcome-MRC Cambridge Stem Cell Institute, Department of Haematology, University of Cambridge, United Kingdom (A.F.D., G.V.).; 7 Now with Department of Cardiology, Osaka City University Graduate School of Medicine, Japan (S.S.).

**Keywords:** cardiotoxicity, clonal hematopoiesis, CRISPR-Cas Systems, DNA damage, heart failure, inflammasome, macrophages

## Abstract

Cancer therapy can be associated with short- and long-term cardiac dysfunction. Patients with cancer often exhibit therapy-related clonal hematopoiesis (t-CH), an aggressive form of clonal hematopoiesis that can result from somatic mutations in genes encoding regulators of the DNA-damage response (DDR) pathway. Gain-of-function mutations in exon 6 of the protein phosphatase Mg2+/Mn2+ dependent 1D (*PPM1D*) gene are the most frequently mutated DNA-damage response gene associated with t-CH. Whether t-CH can contribute to cardiac dysfunction is unknown.

**Objective:**

We evaluated the causal and mechanistic relationships between *Ppm1d*-mediated t-CH and nonischemic heart failure in an experimental system.

**Methods and Results:**

To test whether gain-of-function hematopoietic cell mutations in *Ppm1d* can increase susceptibility to cardiac stress, we evaluated cardiac dysfunction in a mouse model where clonal hematopoiesis-associated mutations in exon 6 of *Ppm1d* were produced by CRISPR-Cas9 technology. Mice transplanted with hematopoietic stem cells containing the mutated *Ppm1d* gene exhibited augmented cardiac remodeling following the continuous infusion of Ang II (angiotensin II). *Ppm1d*-mutant macrophages were impaired in DDR pathway activation and displayed greater DNA damage, higher reactive oxygen species generation, and an augmented proinflammatory profile with elevations in IL (interleukin)-1β and IL-18. The administration of an NLRP3 (NLR family pyrin domain containing 3) inflammasome inhibitor to mice reversed the cardiac phenotype induced by the *Ppm1d*-mutated hematopoietic stem cells under conditions of Ang II–induced stress.

**Conclusions:**

A mouse model of *Ppm1d*-mediated t-CH was more susceptible to cardiac stress. Mechanistically, disruption of the DDR pathway led to elevations in inflammatory cytokine production, and the NLRP3 inflammasome was shown to be essential for this augmented cardiac stress response. These data indicate that t-CH involving activating mutations in *PPM1D* can contribute to the cardiac dysfunction observed in cancer survivors, and that anti-inflammatory therapy may have utility in treating this condition.

## Abstract 2

Supplemental Digital Content is available in the text.

**Meet the First Author, see p 600**

Hematopoietic stem and progenitor cells (HSPC) acquires random somatic mutations during the aging process.^1,2^ Occasionally, these mutations occur in driver genes that provide the HSPC with a competitive growth or survival advantage, and this leads to the expansion of mutant HSPC clones within the bone marrow niche. This condition is prevalent in elderly individuals who lack overt hematologic disorders, and it has been referred to as age-related clonal hematopoiesis and clonal hematopoiesis of indeterminate potential.^3,4^ Epidemiological studies show that mutations in genes encoding epigenetic regulators, including ten-eleven translocation-2 (*TET2*), DNA methyltransferase 3A (*DNMT3A*), additional sex combs like 1 (*ASXL1*), are prevalent in individuals with age-related clonal hematopoiesis.^5–8^ These mutant HSPC give rise to circulating immune cells that harbor the mutant driver gene,^9^ and, in some cases, it has been shown that the mutation alters the function of the progeny immune cells.^1,2^ Although age-associated clonal hematopoiesis in otherwise healthy individuals was initially reported decades ago, only recently has it been appreciated that this condition is associated with an increased risk of all-cause mortality.^5,6,8,10^ While clonal hematopoiesis increases the risk of hematologic cancer, this overall risk is small, and the increased mortality associated with this condition is largely due to an increased incidence of cardiovascular disease (CVD).^6,11–14^ Our laboratory has provided experimental evidence that clonal hematopoiesis attributed to mutations in *TET2*, *DNMT3A*, and *Janus kinase 2* genes (ie, *JAK2*^*V617F*^) can causally contribute to CVD in various animal models by conferring proinflammatory phenotypes to the progeny leukocytes that are derived from the HSPC clones.^15–19^

In contrast to age-related clonal hematopoiesis, therapy-related clonal hematopoiesis (t-CH) refers to the aberrant clonal expansions that are often detected in the blood of cancer survivors.^20,21^ This form of clonal hematopoiesis occurs as a result of the selective pressure that the genotoxic stresses of radiation and chemotherapy exert on the HSPC. Mutations associated with t-CH typically occur in genes that encode regulators of DNA-damage response (DDR) pathway, such as PPM1D (protein phosphatase, Mg2+/Mn2+ dependent 1D), TP53 (tumor protein 53), and checkpoint kinase 2 (CHEK2).^20,22–24^ Of these, *PPM1D*, also referred to as WIP1 (wild-type [WT] p53-induced phosphatase 1), is the most frequently mutated DDR gene associated with t-CH.^20,22^ The *PPM1D* mutations that result in t-CH are localized in exon 6. These mutations result in the stabilization and activation of the PPM1D protein, and they are often referred to as gain-of-function. Although somatic *PPM1D* mutations in blood cells have been detected in patients with therapy-related myelodysplastic syndrome, *PPM1D* is often overlooked as a clonal hematopoiesis driver gene because it is typically not detected in patients with acute myeloid leukemia (AML).^25,26^ However, *PPM1D*-mediated clonal hematopoiesis has recently been reported to be prevalent in patients with chronic ischemic heart failure and associated with poor outcome.^27^ Furthermore, t-CH is predictive of inferior survival and elevated CVD events in patients receiving autologous stem cell transplantation for lymphoma, with *PPM1D* being the most frequently mutated gene in this cohort.^13^

Cancer survivors display increased medium- to long-term risk for nonischemic heart failure.^28^ Notably, the mortality due to CVD in this population is typically greater than that of cancer itself after a 10 year follow-up. To date, the relationship between t-CH and CVD has not been evaluated in an experimental study. Given the mechanistic links between age-related clonal hematopoiesis and CVD, we wondered whether the DDR genes commonly mutated in t-CH could play a role in accelerating the nonischemic heart failure that is prevalent in cancer survivors. Thus, the current study was performed to test whether a causal relationship exists between t-CH and nonischemic heart failure by focusing on activating mutations in exon 6 of the *Ppm1d* gene as a test case.

## Methods

### Data Availability

The detailed methods are available in the Data Supplement. Please see also the Major Resources Table in the Data Supplement. The supporting data are available from the corresponding author upon request.

### Mice

C57BL/6J WT mice (No:000664), B6(C)-Ccr2tm1.1Cln/J (No: 027619), and B6(C)-Gt(ROSA)26Sorem1.1(CAG-cas9*,-EGFP)Rsky/J (No: 028555) were obtained from Jackson Laboratories. Male mice were used for all in vivo experiments, and study protocols were approved by the institutional ACAC at the University of Virginia.

### Lentivirus Production

pLKO5.sgRNA.EFS.tRFP (No. 57823), pLKO5.sgRNA.EFS.GFP (No. 57822), LentiCRISPRv2GFP (No. 82416), psPAX2 (No. 12260), and pMD2.G (No. 12259), were purchased from Addgene. Single gRNA targeting mouse *Ppm1d* (gtcccagctgagatagctag or tggcttaagtcgaagtagcg), nontargeting sgRNA (acggaggctaagcgtcgcaa), or a noncoding sgRNA that targets an intron in the murine *Actb* gene (aggttgctctgacaaccaca)^24^ were subcloned into the BsmB1 restriction enzyme site of the appropriate vector: pLKO5.sgRNA.EFS.tRFP was used for most of the in vivo experiments; pLKO5.sgRNA.EFS.GFP was used for competitive bone marrow transplantation (BMT) experiments; and LentiCRISPRv2GFP was used for cell culture experiments. For *Ppm1d* expression, the mouse cDNA corresponding to 1 to 1326 base pairs, under the spleen focus forming virus (SFFV) promoter, was subcloned into the lentivirus vector.

### Statistical Analyses

All statistical analyses were performed with GraphPad Prism 8 (GraphPad Software Inc, San Diego, CA). The Shapiro-Wilk normality test was used to evaluate data distribution. For normally distributed data with one experimental variable, statistical analyses were performed by parametric analysis: unpaired (2-tailed) Student *t* test for 2 groups and 1-way ANOVA with the Tukey multiple-comparison test for >2 groups. The data with 2 independent variables were evaluated by 2-way ANOVA with post hoc Sidak multiple comparison tests. For these tests, data are presented as mean±SEM. For non-normally distributed data with one experimental variable, statistical analyses were performed by nonparametric analysis: Mann-Whitney *U* test (2-tailed) for 2 groups and Kruskal-Wallis tests with post hoc Dunn multiple comparison tests for >2 groups. For these tests, data are represented as median (minimum, maximum).

## Results

### Generation of a Ppm1d Model of Clonal Hematopoiesis

**Figure 1.**
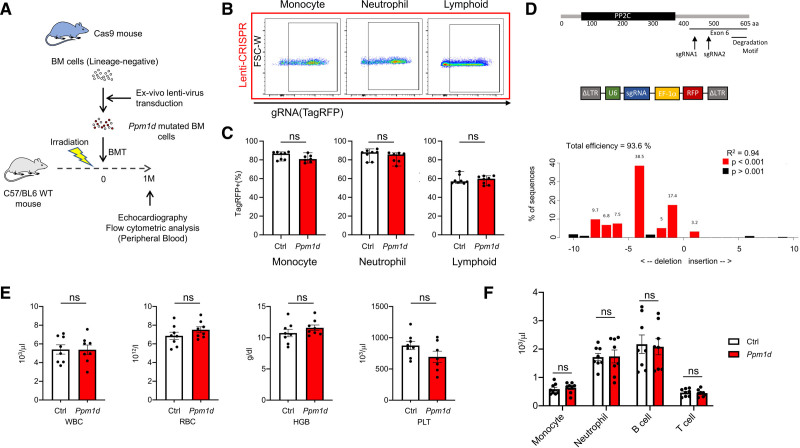
**Ppm1d (protein phosphatase Mg2+/Mn2+ dependent 1D) mutagenesis in hematopoietic stem and progenitor cells (HSPC) and baseline mouse characteristics.A**, Schematic of experimental design. Bone marrow, lineage-negative cells from Cas9 expressing mice were transduced with lentivirus particles expressing single guide RNA (sgRNA)/TagRFP (Tag red fluorescent protein) and delivered to lethally irradiated C57/BL6 wild-type (WT) mice. One month after bone marrow transplantation (BMT), peripheral blood was collected for flow cytometric analysis. **B**, Stable expression of the sgRNA/TagRFP vector at 4 wk post-BMT in monocyte, neutrophil, and lymphoid cells. **C**, Quantitative analysis of TagRFP-positive expression in cell populations derived from hematopoietic stem and progenitor cell (HSPC) transduced with lentiviral vectors encoding control (Ctrl) or *Ppm1d*-targeting sgRNA (n=8 per genotype). Statistical significance was evaluated by Mann Whitney *U* tests. **D**, Tracking of Indels by Decomposition analysis of the TagRFP-positive peripheral blood cells revealing insertions and deletions. **E**, The absolute number of white blood cells (WBC), red blood cells (RBC), hemoglobin (HGB), and platelets (PLT) in both experimental groups (n=8 per genotype). Statistical significance was evaluated by 2-tailed unpaired Student *t* test. **F**, Flow cytometric analysis of the peripheral blood at 4 wk after bone marrow reconstitution with HSPC transduced with control or *Ppm1d*-targeted lentiviral vectors. The absolute number of the major immune cell populations are shown (n=8 per genotype). Statistical significance was evaluated by multiple Student *t* test.

To establish the *Ppm1d* clonal hematopoiesis model, a lentivirus-mediated, CRISPR-Cas9 approach was employed to induce mutations in *Ppm1d* in bone marrow cells before transplantation into recipient mice.^16,29^ The *PPM1D* mutations commonly associated with clonal hematopoiesis are restricted to exon 6 that will promote protein stability and increase in phosphatase activity.^20,23,24^ Thus, bone marrow cells from transgenic mice expressing Cas9 and EGFP from the Rosa26 locus were transfected with lentivirus encoding the TagRFP (Tag red fluorescent protein) reporter gene and a single guide RNA (sgRNA) that targets one of 2 regions in exon 6 of the *Ppm1d* gene (Figure 1A). Control conditions employed a nontarget sgRNA or sgRNA that targets an intron within the *Actb* gene. These cells were then transplanted into groups of WT mice that had been irradiated. Flow cytometric analysis of the peripheral blood at one month after BMT revealed that the efficiency of transfection, as indicated by the percentage of TagRFP positive cells, was ≈80% in monocyte and neutrophil populations and ≈60% in the lymphoid population (Figure 1B and 1C, Figure I in the Data Supplement). Tracking of Indels by Decomposition analysis of the TagRFP-positive peripheral blood mononuclear cells revealed that insertion-deletion (indel) frameshift mutations in *Ppm1d* occurred in 70% to 80% of transplanted cells (Figure 1D). Overall, ≈60% of HSPC had a frameshift mutation in exon 6 of *Ppm1d*, and all the frameshift mutations resulted in the generation of a stop codon. In contrast, there was no evidence of indel mutations in *Ppm1d* in any of the control-transfected groups. Consistent with previous reports,^23,24^ HSPC harboring exon 6 mutations in *Ppm1d* exhibited a competitive advantage in competitive BMT assays under conditions of genotoxic stress (Figure II in the Data Supplement).

Analyses of the blood from mice revealed that the absolute number of peripheral blood cells or hemoglobin levels did not differ between mice transplanted with the mutant cells compared with those transplanted with the control cells (Figure 1E), consistent with the clinical paradigm of clonal hematopoiesis. Furthermore, flow cytometric analysis of the major immune cell subset ratios was not different between the test and control groups (Figure 1F), indicating that exon 6 *Ppm1d* mutations in HSPC does not affect blood cell differentiation. Echocardiographic analysis at 1-month post-BMT did not reveal any overt abnormalities in cardiac function, and cardiac parameters were not different between animals receiving control versus *Ppm1d*-mutant cells (Table I in the Data Supplement). An analysis of immune cells within the heart by flow cytometric analysis revealed no detectable differences in immune cell composition or the degree of chimerism between the control and *Ppm1d*-mutant groups (Figure III in the Data Supplement). Overall, these data indicate that baseline hematologic or cardiac abnormalities are not observed in a mouse model in which a substantial fraction of engrafted HSPC contains mutations in exon 6 of *Ppm1d*.

### Mice With Hematopoietic Ppm1d Mutations Exhibit Augmented Cardiac Remodeling Following Infusion of Ang II

**Figure 2.**
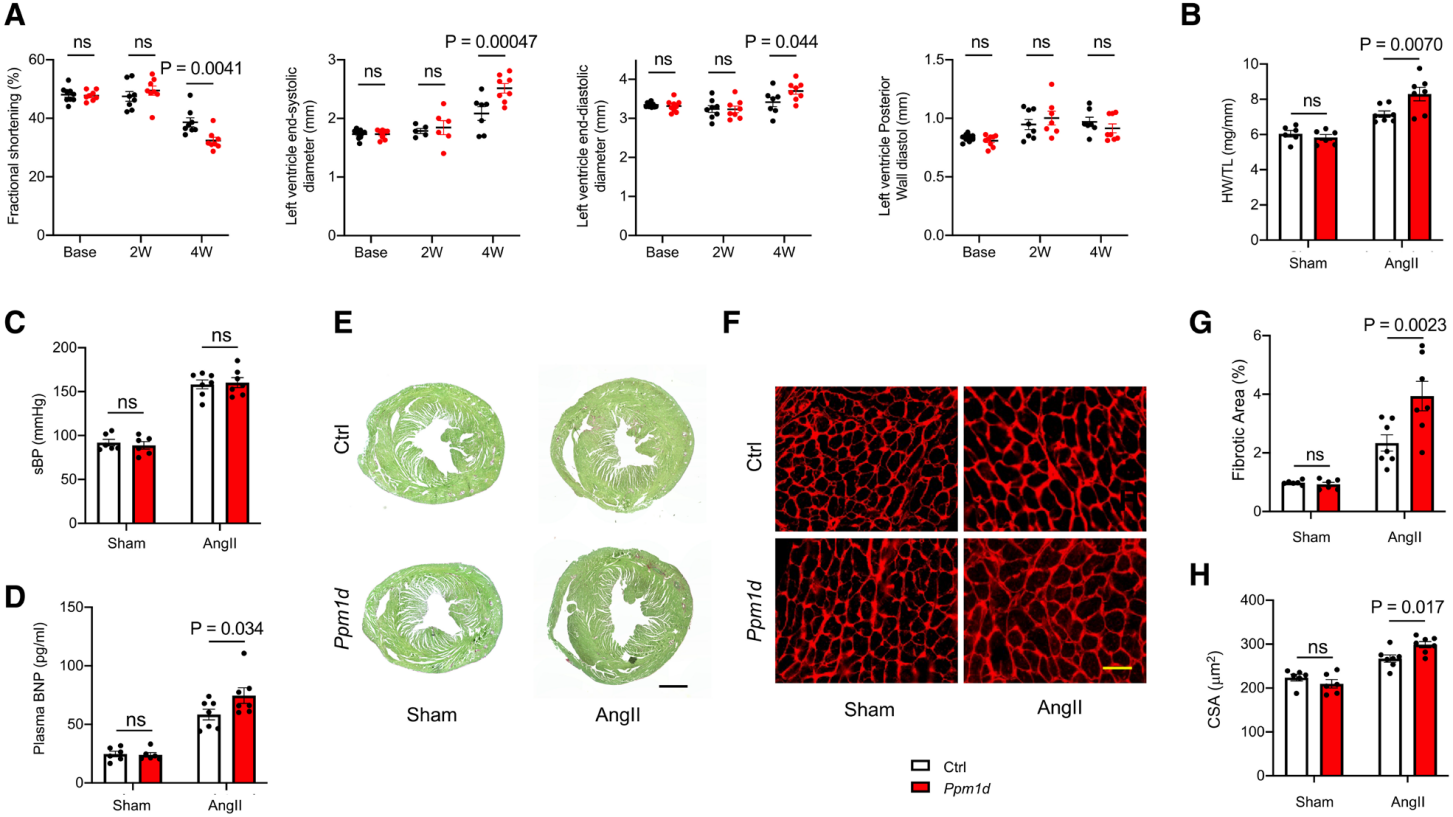
**Ppm1d (protein phosphatase Mg2+/Mn2+ dependent 1D) mutagenesis in hematopoietic stem and progenitor cells (HSPC) leads to greater cardiac pathology in response to Ang II (angiotensin II) infusion.A**, Sequential echocardiographic analysis of mice transplanted with HSPC transduced with lentiviral vectors encoding control (Ctrl) or *Ppm1d*-targeting (n=8 per genotype) at 0 (base) 2 and 4 wk (W) post-Ang II infusion. Statistical significance was evaluated by a 2-way repeated ANOVA with Sidak multiple comparison tests. **B**, Heart weight (HW) adjusted by tibia length (TL) following 4 wk Ang II infusion for experimental groups (n=6 for Sham and n=7 for Ang II). Statistical significance was evaluated by 2-way ANOVA with Sidak multiple comparison tests. **C**, Blood pressure as measured by tail-cuff plethysmography following 4 wk Ang II infusion for the experimental groups (n=6 for Sham and n=7 for Ang II). Statistical significance was evaluated by 2-way ANOVA with Sidak multiple comparison tests. **D**, Serum BNP levels following 28 days of Ang II infusion (n=6 for Sham and n=7 for Ang II). Statistical significance was evaluated by 2-way ANOVA with Sidak multiple comparison tests. **E**, Representative images and analysis of Picrosirius red/Fast Green staining of the heart sections from hearts of control and *Ppm1d*-mutated mice at the end of the study (scale bar=1 mm). Representative images were selected to represent the mean value of each condition. **F**, Representative images of wheat germ agglutinin (WGA) staining of the heart sections from hearts of control and *Ppm1d*-mutated mice at the end of the study (scale bar=50 μm). Representative images were selected to represent the mean value of each condition. **G**, Quantitation of fibrosis in E (n=6 for Sham and n=7 for Ang II). Statistical significance was evaluated by 2-way ANOVA with Sidak multiple comparison tests. **H**, Quantitative analysis of myocyte cross-sectional analysis (n=6 for Sham and n=7 for Ang II). Statistical significance was evaluated by 2-way ANOVA with Sidak multiple comparison tests.

Next, we examined whether *Ppm1d* mutations in HSPC cause mice to be more susceptible to cardiac stress. For the first set of experiments, mice were continuously infused with a supraphysiological dose of Ang II (angiotensin II) for 28 days. Ang II infusion leads to activation of the renin angiotensin-aldosterone system and induces cardiac-remodeling characterized by cardiac fibrosis, hypertrophy, and dysfunction.^30^ Analysis of these end points after infusion with Ang II revealed greater cardiac remodeling in the mice that harbored HSPC with the *Ppm1d* mutation compared with the control nontargeted sgRNA group (Figure 2). Echocardiographic analysis revealed a time-dependent decrease in fractional shortening and an increase in left ventricle end-diastolic and end-systolic diameters in mice that received the *Ppm1d*-mutant cells at the 4-week timepoint after Ang II administration (Figure 2A). Heart weights were significantly greater in the *Ppm1d*-mutant group in the absence of differences in blood pressure between experimental groups as measured by the tail-cuff plethysmography (Figure 2B and 2C). Autopsy analyses did not reveal aortic dissections/aneurysms in either experimental group. Plasma BNP, a heart failure marker, was significantly increased in the *Ppm1d*-mutant group (Figure 2D). Picrosirius red/Fast Green staining of the heart showed greater levels of fibrosis in the *Ppm1d*-mutant group (Figure 2E and 2G), and an increase cardiomyocyte cross-sectional area size that is consistent with greater cardiac hypertrophy (Figure 2F and 2H).

Three independent sets of experiments were performed to further test the hypothesis that exon 6 mutations in *Ppm1d* promote Ang II-induced cardiac remodeling. First, the time course was extended to 2 months of Ang II infusion by the implantation of a second osmotic pump (Figure IV in the Data Supplement). Compared with the 1-month timepoint, the later time point revealed greater cardiac remodeling by the continuous infusion of Ang II and a greater separation of the data between test and control conditions. In a second set of experiments, *Ppm1d* gene editing was directed by the lentivirus-mediated delivery of a second, independent sgRNA that was also predicted to have at least 4 mismatches with any other coding region in the genome (Figure V in the Data Supplement). Compared with the initial sgRNA targeting the *Ppm1d* gene, there was a nearly identical impact on Ang II-induced cardiac remodeling, indicating that the enhanced cardiac pathology is not due to off-target effects of the sgRNA. Finally, we compared the *Ppm1d*-targeting sgRNA with a control sgRNA that targets an intron in the *Actb* gene (Figure VI in the Data Supplement). The *Actb*-targeting sgRNA species did not detectably augment the hypertrophic response and, compared with this sgRNA control, the *Ppm1d*-targeting condition promoted robust cardiac remodeling in response to Ang II stimulation. Collectively, these data indicate that mice with the gain-of-function, exon 6 *Ppm1d* mutations in hematopoietic cells have normal cardiac function at baseline, but display greater cardiac remodeling and dysfunction following Ang II infusion.

### Infiltrating Monocytes and Macrophages Have an Essential Role in Cardiac Remodeling Response to Ang II Infusion

**Figure 3.**
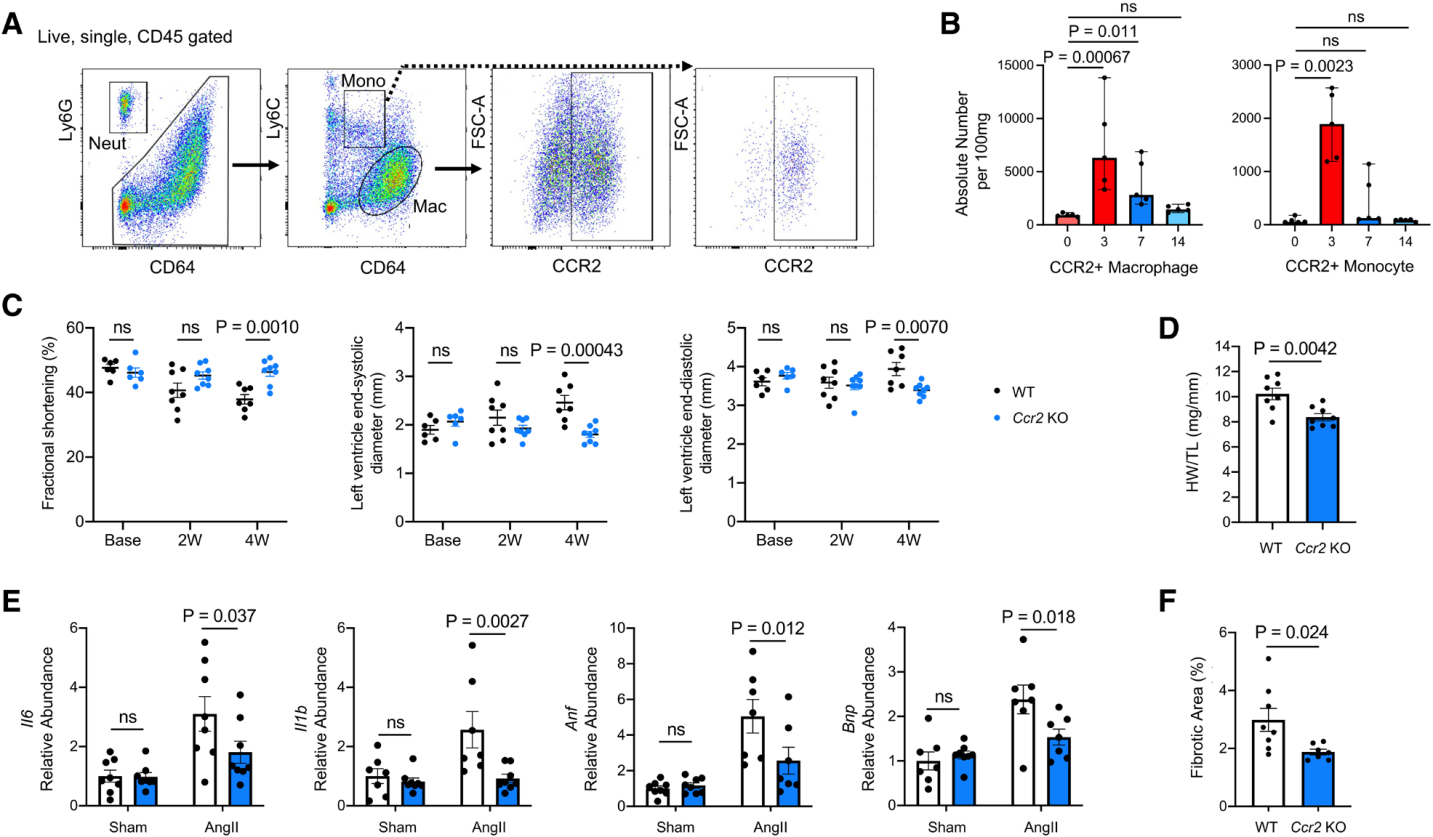
**CCR2 (C-C motif chemokine receptor 2)-positive immune cell involvement in Ang II (angiotensin II) induced cardiac remodeling.A**, Representative flow cytometry analysis of digested hearts after Ang II infusion. Cells were defined as neutrophils (Neut; CD45^+^Ly6G^+^CD64^−^), monocytes (Mono; CD45^+^Ly6G^−^CD64^int^Ly6C^hi^), or macrophages (Mac; CD45^+^Ly6G^−^CD64^+^Ly6C^low^). **B**, Quantitation of flow cytometry analysis of immune cells in the heart at 0, 3, 7, 14 days after Ang II infusion (n=5 per timepoint). The absolute number per 100 mg is shown. Statistical significance was evaluated by Kruskal-Wallis tests with Dunn multiple comparison tests. **C**, Sequential echocardiography analysis of wild-type (WT) and *Ccr2*-deficient (*Ccr2*-KO) mice (n=8 per genotype) at baseline (base) and 2 and 4 wk (W) post-Ang II infusion. Statistical significance was evaluated by 2-way repeated ANOVA with Sidak multiple comparison tests. **D**, Heart weight (HW) adjusted by tibia length (TL) at the end of the study (n=8 per genotype). Statistical significance was evaluated by 2-tailed unpaired Student *t* test. **E**, Gene expression analysis in hearts from WT and *Ccr2*-KO mice at 4 wk after infusion of saline or Ang II (n=8 per group). Statistical significance was evaluated by 2-way ANOVA with Sidak multiple comparison tests. **F**, Quantitation of fibrosis (n=8 per genotype). Statistical significance was evaluated by 2-tailed unpaired Student *t* test.

Since the *Ppm1d* mutation was introduced into the HSPC fraction that repopulates the bone marrow, multiple leukocyte progeny derived from these HSPC will express the edited gene. Furthermore, because relatively little is known about the roles of immune cells in nonischemic heart failure, we examined the content of immune cells in the heart over time following the initiation of Ang II infusion to develop a better understanding of the leukocyte populations that could be contributing to the development of cardiac dysfunction in this t-CH model. Hearts were analyzed by flow cytometry at 0, 3, 7, and 14 days following the initiation of Ang II to characterize the dynamics of the cardiac immune cell content and infiltration over the course of cardiac hypertrophy in WT mice (Figures VII and VIII in the Data Supplement). We observed a significant increase in the absolute numbers of cardiac monocytes (defined as CD45^+^CD64^int^Ly6C^hi^), macrophages (defined as CD45^+^CD64^+^Ly6C^lo^), and neutrophil (defined as CD45^+^CD64^−^Ly6G^+^) at 3 days. When monocytes and macrophages were further gated for expression of the CCR2 (C-C motif chemokine receptor 2),^31^ it was revealed that the CCR2+ monocyte and macrophage populations displayed the greatest elevation in the heart in response to Ang II stimulation (Figure 3A and 3B). In contrast to these changes in myeloid cells, little or no changes were observed in the absolute numbers of B or T cells within the heart in response to Ang II stimulation (Figure VIII in the Data Supplement).

To explore the functional role of monocytes and macrophages in this system, the cardiac stress response to Ang II was evaluated in mice deficient in CCR2 (*Ccr2*-KO), that is required for monocyte mobilization from bone marrow. Echocardiographic analysis revealed a time-dependent decrease in fractional shortening and an increase in left ventricle end-systolic diameter in the control mice, but these changes were abrogated in the *Ccr2*-KO group (Figure 3C). Compared with WT mice, heart weight was significantly lower in the *Ccr2*-KO group after 28 days of Ang II infusion (Figure 3D). Analysis of transcript expression in the isolated hearts revealed that transcript expression of the proinflammatory cytokines *Il6* and *Il1b* and the heart failure markers *Anf* and *Bnp* were significantly lower in *Ccr2*-KO mice (Figure 3E). Consistent with this reduction in inflammation, Picrosirius red/Fast Green staining of the heart revealed less extensive fibrosis in the *Ccr2*-KO mice (Figure 3F).

While *Ccr2*-KO mice are protected from the effects of Ang II infusion, it is unknown whether this effect is mediated by the loss of CCR2 in bone marrow cells or endothelial cells, fibroblasts, myocytes, etc that also express this gene.^32^ Thus, to analyze this in greater detail, lethally irradiated *Ccr2*-KO mice were transplanted with either WT or *Ppm1d* mutant HSPC and then treated with the continuous Ang II infusion. Following transplantation, *Ccr2*-KO mice acquired susceptibility to Ang II–induced cardiac remodeling, and transplantation with the *Ppm1d*-mutant HSPC conferred a worse cardiac remodeling phenotype compared with the mice with WT HSPC (Figure IX in the Data Supplement). Collectively, these data document the importance of infiltrating, bone marrow-derived monocytes/macrophages in this cardiac remodeling response, and they further corroborate cardiac remodeling phenotype that is conferred by the *Ppm1d* mutant HSPC. Thus, the role of *Ppm1d* mutations in the macrophage population was assessed further.

### Ppm1d-Mutant Macrophages Exhibit DDR Pathway Impairment and an Augmented Proinflammatory Profile

**Figure 4.**
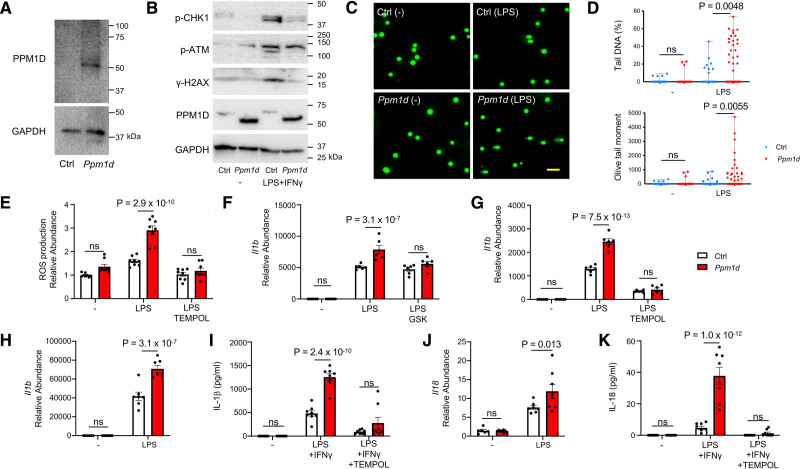
**Ppm1d (protein phosphatase Mg2+/Mn2+ dependent 1D)-mutant macrophages exhibit impaired DNA-damage response (DDR) pathway activity and an augmented proinflammatory profile.A**, PPM1D expression assessed by immunoblot analysis in bone marrow derived macrophages. **B**, CHK1 (checkpoint kinase 1), ATM (ataxia telangiectasia mutated), and H2AX (γH2A histone family member X) phosphorylation (P) and PPM1D protein expression evaluated by immunoblot analysis in J774.1 cells treated with lentivirus with control (Ctrl) or *Ppm1d*-tartgetted sgRNA. Cells were stimulated with 20 ng/mL lipopolysaccharide (LPS)/50 ng/mL IFN (interferon)-γ for 6 h **C**, Representative images from the comet assay comparing control (Ctrl) and *Ppm1d*-targetted groups in the presence and absence of LPS/IFN-γ (scale bar=100 μm). Representative images were selected to represent the mean value of each condition. **D**, Quantitative analysis of the comet assay for the different experimental groups by assessing the Tail DNA (%) and the olive tail moment (n=118, 89, 79, 97 for each group, respectively). Statistical significance was evaluated by Kruskal-Wallis tests with post hoc Dunn multiple comparison tests. **E**, Assessment of reactive oxygen species (ROS) production by dichlorofluorescein (DCF) assay of control (Ctrl) and *Ppm1d*-mutated J774.1 cells at 6 h after stimulation with 10 ng/mL LPS in the presence or absence of 10 mmol/L of 4-hydroxy-2,2,6,6-tetramethylpiperidin-1-oxyl (TEMPOL) (n=8 per group). Statistical significance was evaluated by 2-way ANOVA with Sidak multiple comparison tests. **F** and **G**, Gene expression analysis of control (Ctrl) and *Ppm1d*-mutated J774.1 cells at 6 h after stimulation with 10 ng/mL LPS with/without 3.0 µmol/L of PPM1D inhibitor GSK2830371 (GSK) or 10 mmol/L of TEMPOL (n=6 per group). Statistical significance was evaluated by 2-way ANOVA with Sidak multiple comparison tests. **H** and **J**, Gene expression analysis of bone marrow-derived macrophages isolated from mice receiving control (Ctrl) or *Ppm1d*-mutated HSPCs following stimulation with 10 ng/mL LPS for 6 h (n=7 mice per group). Statistical significance was evaluated by 2-way ANOVA with Sidak multiple comparison tests. **I** and **K**, ELISA analysis of IL (interleukin)-1β and IL-18 in the supernatant of bone marrow-derived macrophages isolated from mice receiving control (Ctrl) or *Ppm1d*-mutated HSPCs. Bone marrow-derived macrophage was stimulated with 20 ng/mL LPS/50 ng/mL IFN-γ in the presence or absence of 10 mmol/L of TEMPOL for 6 h in combination with a final 30-min incubation of 10 mmol/L ATP (n=8 mice per group.) Statistical significance was evaluated by 2-way ANOVA with Sidak multiple comparison tests.

PPM1D expression was assessed in myeloid cells isolated from mice transplanted with *Ppm1d*-mutant HSPC. Western blot analysis revealed robust expression of the stabilized (ie, truncated) form of PPM1D in bone marrow derived macrophages (Figure 4A, Figure V in the Data Supplement). Subsequent studies assessed the role of C-terminal *Ppm1d* mutations in controlling the DDR pathway and cytokine expression in the both in murine J774.1 monocyte/macrophage cell line and bone marrow-derived macrophages. PPM1D is a serine-threonine protein phosphatase that dephosphorylates several substrates, including ATM (ataxia telangiectasia mutated) serine/threonine kinase, CHK1 (checkpoint kinase 1), and γH2AX (γH2A histone family member X) and negatively regulates the DDR pathway.^33^ Thus to examine the effect of the *Ppm1d* truncation mutation on the DDR pathway, we generated a murine J774.1 cell line that expresses the exon 6-mutated forms of *Ppm1d* using the CRISPR-Cas9 approach described above. The nature of the CRISPR/Cas9-edited mutations in exon 6 of *Ppm1d* is shown in Figure X in the Data Supplement. These mutations led to a marked increase in the PPM1D protein expression, but they had little or no impact on the phosphorylation of ATM, CHK1, or γH2AX under nonstimulated conditions (Figure 4B). However, the phosphorylation of these proteins was suppressed by the exon 6 mutations in *Ppm1d* in cells stimulated with LPS/IFN-γ (interferon-γ). Whereas it has been reported that the complete germline ablation of *Ppm1d* can enhance inflammation through p65-NFκB activation,^34^ the gain-of-function *Ppm1d* mutations examined here had little or no effect on the phosphorylation levels of p65 NFκB or p38 MAPK under these same conditions (Figure XI in the Data Supplement).

To examine the functional consequences of *Ppm1d* gene editing on the DDR pathway function, J774.1 cells were exposed to LPS to induce DNA-damage and inflammatory cytokine production through the activation of TLR4 (toll-like receptor 4) signaling.^35^ The comet assay, which detects cellular DNA-damage, revealed greater DNA fragmentation in the LPS-stimulated *Ppm1d*-mutant cells compared with LPS-treated control cells (Figure 4C and 4D). These observations were corroborated using LPS-stimulated bone marrow-derived macrophages isolated from mice that were transplanted with WT or *Ppm1d*-mutated cells (Figure XII in the Data Supplement).

To extend these findings, DNA damage was assessed in J774.1 cells in the presence or absence of the genotoxic agent doxorubicin under the following conditions: (1) untreated cells (nonmodified), (2) lentivirus/Cas9/sgRNA that does not target a DNA locus (nontargeted), (3) lentivirus/Cas9/sgRNA treatment that targets an intron of the *Actb* gene (noncoding; Figure VI in the Data Supplement), (4) lentivirus/Cas9/sgRNA treatment that targets exon 6 of PPM1D gene (*Ppm1d*), and (5) treatments with a lentivirus vector that overexpresses the truncated form of PPM1D (*Ppm1d* O/E) and lacks the CRISPR system components (Figure XIII in the Data Supplement). In the absence of stimulation, there was little or no DNA damage, suggesting that the DDR is not altered at baseline (Figure XIV in the Data Supplement). After stimulation with doxorubicin, there was detectable DNA damage in all experimental groups, but the DNA damage in *Ppm1d* gene-edited condition was significantly greater than that of the control groups. Consistent with findings in the mouse model, there was no difference among the different control groups regardless of whether they were not treated, transduced with a nontargeting sgRNA or transduced with a sgRNA that targets the intron of the *Actb* gene (Figure XIV in the Data Supplement). Collectively, these assays revealed no evidence of DDR activation by the CRISPR system per se. However, the exon 6 mutant form of *Ppm1d*, produced either by CRISPR gene editing or by the direct expression of this mutant form, led to suppression of the DDR and substantially greater DNA damage when cells were stimulated with a genotoxic agent.

The immune-regulatory properties of the gain-of-function, exon 6 *Ppm1d*-mutations have not been described previously. Thus, LPS-stimulated, mutant and control J774.1 cells were evaluated for cytokine and chemokine expression, and reactive oxygen species (ROS) production by the dichlorofluorescein assay because DNA damage can activate ROS production.^36^ After stimulation with LPS, ROS production was increased in both WT and *Ppm1d*-mutant cells, but the increase in ROS was markedly greater in *Ppm1d*-mutant condition (Figure 4E). Treatment with 4-hydroxy-2,2,6,6-tetramethylpiperidin-1-oxyl (TEMPOL) suppressed LPS-induced ROS production in both control and *Ppm1d*-mutant cells, and there was no detectable difference between mutant and control cells after treatment. The *Ppm1d*-mutant cells also displayed higher levels of *Il1b*, *Il6*, and *Cxcl2* transcripts following stimulation with LPS (Figure 4F and 4G, Figure XV in the Data Supplement). Higher levels of *Il1b* were also observed in J774.1 cells that overexpress the truncated form of PPM1D in the absence of the CRISPR system reagents (Figure XVI in the Data Supplement). To further investigate this inflammatory response, cells were incubated with the PPM1D inhibitor GSK2830371 or the antioxidant compound TEMPOL. As expected, the GSK2830371 compound had little or no effect on the expression of *Il1b* in WT cells, but it markedly inhibited *Il1b* expression in the *Ppm1d*-mutant cells (Figure 4F). TEMPOL treatment also suppressed LPS-induced *Il1b* expression in WT and *Ppm1d*-mutant cells and eliminated the difference in *Il1b* expression between the mutant and control conditions (Figure 4G). To corroborate these findings, cytokine expression levels were analyzed in bone marrow-derived macrophages isolated from mice transplanted with *Ppm1d*-mutant or WT cells (Figure 4H through 4K). LPS-stimulated macrophages from the *Ppm1d*-mutant group showed elevated expression of both *Il1b* and *Il18* transcripts (Figure 4H and 4J), and an ELISA analysis revealed a significant increase in the secretion of IL (interleukin)-1β and IL-18 following LPS/IFN-γ/ATP treatment in the *Ppm1d*-mutant macrophages (Figure 4I and 4K). TEMPOL treatment attenuated the elevated secretion of IL-1β and IL-18 in both control and the *Ppm1d*-mutant bone marrow-derived macrophages, and there was no detectable difference between these 2 groups in the TEMPOL-treated cells (Figure 4I and 4K). Collectively, these results suggest that activating mutations in the C-terminal exon of *Ppm1d* lead to the dephosphorylation of PPM1D substrates and inactivation of the DDR pathway. In turn, these events will result in the accumulation of DNA-damage, ROS production, and a proinflammatory phenotype.

### The Inflammasome Has an Essential Role in the Ppm1d-Mediated Cardiac Dysfunction

**Figure 5.**
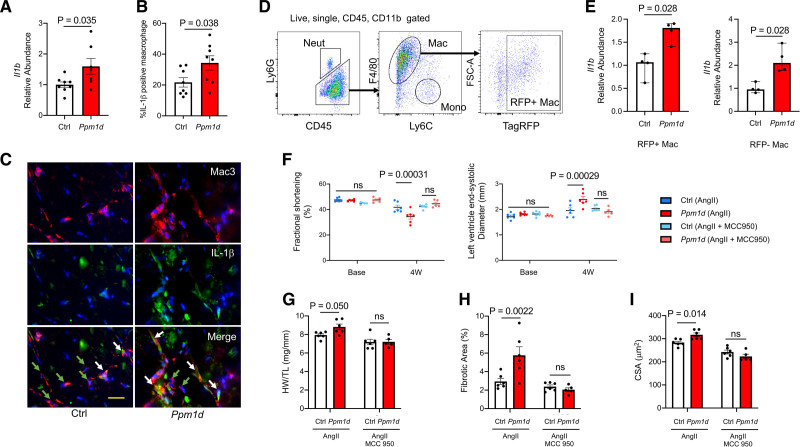
**Involvement of IL (interleukin)-1β and the NLRP3 inflammasome in cardiac dysfunction mediated by Ppm1d (protein phosphatase Mg2+/Mn2+ dependent 1D)-mutated hematopoietic stem and progenitor cells (HSPC).A**, *Il1b* gene expression analysis of hearts from mice transplanted with HSPC transduced with lentiviral vectors encoding control (Ctrl) or *Ppm1d*-targeting single guide RNAs (sgRNAs), at 4 wk after Ang II (angiotensin II) infusion (n=8 for Ctrl and n=7 for *Ppm1d* group). Statistical significance was evaluated by 2-tailed unpaired Student *t* test. **B**, Quantification of IL-1β positive macrophages in heart tissues at the termination of the experiment. (n=8 per group). Statistical significance was evaluated by 2-tailed unpaired Student *t* test. **C**, Representative images of IL-1β immunofluorescence staining (green) in Mac3-positive macrophages (red) from control (Ctrl) and *Ppm1d*-mutated mice. Scale bars=20 μm. Representative images were selected to represent the mean value of each condition. **D**, Flow cytometry gating strategy for cardiac macrophage cells. Cells are defined as: RFP+ macrophage (RFP+ Mac; CD45^+^CD11b^+^Ly6G^−^F4/80^+^Ly6C^low^RFP+), RFP^−^ macrophage (RFP^−^ Mac; CD45^+^CD11b^+^Ly6G^−^F4/80^+^Ly6C^low^RFP^−^). Transduced cells are TagRFP positive. **E**, *Il1b* gene expression analysis of sorted RFP+ and RFP^−^ cardiac macrophages from mice transplanted with HSPCs transduced with lentiviral vectors encoding control (Ctrl) or *Ppm1d*-targeting sgRNAs, at 3 days after Ang II infusion (n=4 per group). Statistical significance was evaluated by Mann Whitney *U* tests. **F**, Sequential echocardiography analysis of Ang II-treated mice transplanted with control (Ctrl) and *Ppm1d*-mutated HSPC in the presence or absence of co-infusion with MCC950 (MCC) at baseline and at the end of the study period (n=6 for Ctrl-Ang II, *Ppm1d*-Ang II, and Ctrl-Ang II+MCC950, n=5 for *Ppm1d*-Ang II+MCC950). Statistical significance was evaluated by 2-way repeated ANOVA with Sidak multiple comparison tests. **G**, Heart weight (HW) adjusted by tibia length (TL) for the experimental groups described in **E** after the 4-week study period (n=6 for Ctrl-Ang II, *Ppm1d*-Ang II, and Ctrl-Ang II + MCC950, n=5 for *Ppm1d*-Ang II+MCC950). Statistical significance was evaluated by 2-way ANOVA with Sidak multiple comparison tests. **H**, Cardiac fibrosis for the experimental groups described in **E** (n=6 for Ctrl-Ang II, *Ppm1d*-Ang II, and Ctrl-Ang II+MCC950, n=5 for *Ppm1d*-Ang II+MCC950). Statistical significance was evaluated by 2-way ANOVA with Sidak multiple comparison tests. **I**, Analysis of hypertrophy in cardiac sections determined by myocyte cross-sectional area. Statistical significance was evaluated by 2-way ANOVA with Sidak multiple comparison tests. (n=6 for Ctrl-Ang II, *Ppm1d*-Ang II, and Ctrl-Ang II+MCC950, n=5 for *Ppm1d*-Ang II+MCC950).

Additional experiments focused on the upregulation of *Il1b* in the *Ppm1d*-mutant condition because this cytokine has previously been implicated clonal hematopoiesis and Ang II–mediated cardiac pathologies.^15,17,37^ Analysis of transcript expression in hearts revealed that the expression of *Il1b* was significantly higher in mice that had been transplanted with *Ppm1d*-mutant bone marrow cells (Figure 5A). To assess the major sources of IL-1β and NLRP3 (NLR family pyrin domain containing 3) expression in the heart, fibroblasts (CD45^−^Ter119^−^CD31^−^PDGFRα^+^Sca1^+^), endothelial cells (CD45^−^CD11b^−^CD31^+^Ter119^−^), and myeloid cells (CD45^+^CD31^−^CD11b^+^) were sorted from WT mice at 3 days after treatment with Ang II. The quantitative polymerase chain reaction analysis showed that all cell types express *Il1b* and *Nlrp3*; however, their expression is vastly greater in the myeloid cells compared with the other cell types (Figure XVII in the Data Supplement). Immunofluorescence staining of heart tissue also revealed greater numbers of IL-1β-positive macrophages compared with control hearts from mice that had been treated with Ang II (Figure 5B and 5C). Very few dead macrophages were identified in the myocardium as assessed by TUNEL staining, and there was no difference between mice transplanted with either WT or *Ppm1d*-mutant bone marrow (Figure XVIII in the Data Supplement). To extend these findings, cytokine expression levels were analyzed in macrophages sorted from mice transplanted with *Ppm1d*-mutant or WT cells after 3 days of Ang II infusion. Indicative of a noncell autonomous effect of clonal hematopoiesis that has been noted by others,^38^ both RFP+ (*Ppm1d*-mutant) and RFP− (WT) macrophages expressed elevated levels of *Il1b* transcript in the mice transplanted with *Ppm1d*-mutant cells (Figure 5D and 5E). Further analyses revealed that the heart tissue from Ang II–treated mice transplanted with either WT or *Ppm1d*-mutant HSPCs did not differ in the absolute numbers of monocytes, macrophages or neutrophils within the heart between the 2 experimental groups (Figure XIX in the Data Supplement). In addition, the degree of chimerism of TagRFP-positive cells were similar to what was observed in the peripheral blood for each immune cell subset. Collectively, these data suggest that the *Ppm1d*-mutated HSPC give rise to proinflammatory cardiac myeloid cells, but these conditions lead to little or no effect on the infiltration of immune cells to the heart.

IL-1β is synthesized as a precursor protein (pro-IL-1β) and requires cleavage by the NLRP3 inflammasome for activity. To assess the functional significance of NLRP3 inflammasome in the augmented cardiac pathology observed in mice transplanted with *Ppm1d* mutant cells, the inflammasome inhibitor MCC950 was administered at the time of Ang II infusion. Co-infusion of MCC950 for 28 days resulted in protection against the enhanced Ang II–induced cardiac remodeling in the mice that had received *Ppm1d*-mutant HSPC (Figure 5F through 5I). The effect of MCC950 treatment was quantitatively greater in the *Ppm1d*-mutant group, and this treatment eliminated the statistical differences in the echocardiographic parameters of left ventricle end-systolic diameters and fractional shortening between mice transplanted with control cells and *Ppm1d*-mutant cells (Figure 5F). Consistent with these data, MCC950 had similar protective effects on the parameters of heart weight to tibia length ratio (Figure 5G), the extent of fibrosis (Figure 5H), and the increase in myocyte CSA at the 28-day time point (Figure 5I). For all of these parameters, treatment with MCC950 inhibited the pathological remodeling in the *Ppm1d*-mutant group and eliminated the differences in these parameters between mice transplanted with control and mutant cells. Overall, these data suggest that the greater cardiac remodeling and dysfunction observed in mice receiving *Ppm1d*-mutant HSPC is likely due to an augmentation of NLRP3 inflammasome-mediated cytokine production under conditions of Ang II infusion.

## Discussion

While the prevalence of clonal hematopoiesis increases with age, genotoxic agents, such as radiation and chemotherapy, have been associated with t-CH in younger individuals who are cancer survivors.^20,22^ Approximately 30% of cancer survivors have at least one detectable clonal event in their white blood cells that display mutations in a set of drivers that include DDR genes.^22^ The DDR gene *PPM1D*, with activating mutations in exon 6, is the most highly enriched t-CH driver gene.^20,22^ Recent studies show that HSPC harboring the gain-of-function *PPM1D*-mutant clones have the ability to avoid cell death induced by DNA-damage.^23,24^ Thus, these mutations can confer a competitive survival advantage to HSPC under conditions of genotoxic stress and result in the clonal expansion of their progeny leukocytes in blood.

It is widely appreciated that radiation or chemotherapy can contribute to the development of nonischemic heart disease in cancer survivors.^28^ While epidemiological data support an association between t-CH and CVD in cancer survivors,^13^ causal and mechanistic relationships have never been evaluated. Thus, we developed a mouse model to examine whether somatic hematopoietic mutations in exon 6 of *Ppm1d* can increase the heart’s susceptibility to stress. This model specifically focused on the impact of t-CH on cardiac dysfunction that can occur in cancer survivors long after their therapy has ended, rather than on the shorter term adverse cardiac effects of cancer therapy in patients with preexisting clonal hematopoiesis. It was observed that mice transplanted with *Ppm1d*-mutant cells exhibit greater pathological cardiac remodeling following Ang II infusion. The results of this experimental study are supported by a recent study that found an association between *PPM1D*-mediated clonal hematopoiesis and worse outcome in chronic ischemic heart failure.^27^ Collectively, these findings raise the possibility that *PPM1D*-mediated t-CH may be a factor that contributes to the development of late-onset heart failure in cancer survivors.

Mechanistically, macrophages with exon 6 *Ppm1d* mutations exhibited higher expression of IL-1β and IL-18. As there were no detectable changes in the absolute numbers of cardiac monocytes and macrophages between the experimental groups, it would appear that the exon 6 *Ppm1d* mutation promotes a proinflammatory macrophage phenotype rather than influencing the cardiac infiltration of myeloid cells. Consistent with these observations, editing the *Ppm1d* gene at exon 6 in bone marrow-derived macrophages or the monocyte/macrophage J774.1 cell line increased the transcription and secretion of the IL-1β and IL-18 in response to stimulation. To test for the involvement of the NLRP3 inflammasome in cardiac remodeling in the *Ppm1d*-mutant condition, mice were administered the NLRP3 inflammasome inhibitor MCC950 that prevents the processing of IL-1β and IL-18 to their active forms. This treatment effectively reversed the cardiac phenotype caused by the transplantation of the *Ppm1d*-mutated HSPC, suggesting that inflammasome activation and elevated cytokine production was critical for the enhanced pathological cardiac phenotype.

The immune-regulatory properties of the activating, exon 6 *Ppm1d*-mutations have not been described previously. With regard to clonal hematopoiesis, our prior studies have shown that mutations in the *Tet2* driver gene activate the inflammasome and elevate IL-1β through a HDAC (histone deacetylase)-dependent mechanism.^15^ In contrast, it appears that activating mutations in *Ppm1d* promote IL-1β (and IL-18) expression via a mechanism that involves modulation of the DDR response. Double strand DNA (dsDNA) breaks occur naturally as a result of endogenous metabolic and DNA replicative processes, and this damage is accelerated by exposure to genotoxic agents. In response to this stress, cells mount a canonical DDR to repair the DNA breaks and protect the integrity of the genome via the activation of TP53. In turn, TP53 induces the expression of PPM1D, comprising a negative feedback loop that inactivates the DDR through the inactivating dephosphorylation of TP53, CHK1 and 2, ATM, and γH2AX.^33^ The t-CH mutations in exon 6 of *PPM1D* are activating and will suppress the DDR. As shown here, exon 6 mutations of *Ppm1d* lead to a greater degree of DNA damage and greater inflammation in stimulated macrophages. Treatment with the ROS scavenger TEMPOL inhibited the induction of *Il1b* and *Il18* and diminished their protein products in *Ppm1d*-mutant cells, suggesting that ROS contributes to cytokine production under these conditions. Consistent with the notion that inflammation is triggered by DNA damage, it is widely recognized cytoplasmic dsDNA triggers the formation and activation of the AIM2 (absent in melanoma 2) innate immune sensor that mediates the assembly and activation of the inflammasome.^39^ Cytosolic dsDNA is also sensed by the cGAS (cyclic GMP-AMP synthase)-Stimulator of Interferon Genes pathway which further promotes activation of the NLRP3 and AIM2 inflammasomes in macrophages.^40^ Supporting these observations, macrophage inflammasome activity is promoted by the diminished activity of other DDR components including the DNA-damage repair kinase ATM^41^ and the Nbs1 (Nijmegen breakage syndrome 1) proteins that are necessary for the detection of DNA damage.^42^ While we cannot rule out any of these parallel mechanisms, our experimental findings are consistent with the hypothesis that Ppm1d stabilization promotes ROS production through the suppression of ATM and other components of the DDR pathway (Figure 4B), thereby leading to activation of the inflammasome in an ROS-dependent manner (Figure 4I and 4K). Overall, the mechanistic findings reported here are consistent with prior studies that have linked the suppression of the DDR pathway to ROS generation and ROS generation to elevated inflammation.^43–45^

It is reported that germline *Ppm1d*-knockout (loss-of-function) mice display augmented inflammatory phenotypes that results from abnormalities in B cell, T cell, and neutrophil differentiation.^33^ In contrast to these findings, this study and another^23^ demonstrate that mice expressing exon 6-specific *Ppm1d* mutations in the hematopoietic system do not display detectable abnormalities in blood cell differentiation. It has also been reported that HeLa cells that lack functional PPM1D display increased inflammatory cytokine transcript expression via increased p65 NF-κB phosphorylation.^34^ However, we did not observe a detectable effect of the exon 6-specific *Ppm1d* mutations on p65 NF-κB in this system, perhaps because this regulation is dependent on the specific cell type.^46^ Thus, the proinflammatory properties of the exon 6-mutated, *Ppm1d* gain-of-function mice represents a previously unrecognized phenotype that provides insights about the mechanisms by which *PPM1D*-mediated t-CH mutations contribute to CVD.

The current study also provides evidence supporting the critical role that myeloid cells play in nonischemic heart failure.^47,48^ Previous work has documented the expansion of the cardiac macrophage pool during the early phases of Ang II infusion,^49^ but the functional role of monocyte derived-macrophages in this model is controversial.^50,51^ Here, it is shown that the cardiac remodeling response to Ang II stimulation is highly dependent on the participation of bone marrow-derived CCR2+ monocytes and macrophages. Among various leukocyte populations, CCR2+ cells displayed the greatest elevation in the heart in response to Ang II stimulation, and in contrast to a prior report,^51^ mice deficient in this chemokine receptor were found to be markedly protected from Ang II-induced cardiac remodeling. Furthermore, when the *Ccr2*-KO mice were transplanted with *Ccr2*-positive HSPC, the pathological actions Ang II on the heart were restored and the cardiac pathology was enhanced by the exon 6 mutations in *Ppm1d*. Collectively, these data indicate that bone marrow-derived myeloid cells are major contributors to the development of nonischemic heart failure, and that inflammasome overactivation in this cellular compartment is a critical feature of the exaggerated pathological responses under the conditions of *PPM1D*-mediated t-CH.

While our prior work has shown that different mutant driver genes can confer distinct phenotypes to their progeny leukocytes within the clone,^1^ a growing body of experimental^15–19^ and epidemiological,^11,52^ evidence suggests that activation of IL-1β or IL-6 expression represents a common feature shared by many clonal hematopoiesis driver genes. The experimental findings reported here for exon 6 mutations in *Ppm1d* further strengthen the connection between overactivation of cytokine signaling and clonal hematopoiesis-mediated cardiovascular pathology. In this regard, there is growing evidence that proinflammatory cytokines affect HSPC behavior and alter their bone marrow niche.^53^ It has been reported that Tet2-deficiency also confers a selective advantage to HSPC in response to chronic proinflammatory signals.^54^ Thus, it is tempting to speculate that many clonal hematopoiesis driver genes will share this feature of promoting inflammation, particularly via IL-6 and IL-1β, while shielding the mutant HSPC from the suppressive effects of chronic cytokine overactivation. In other words, the upregulation IL-6 and IL-1β may not only be a common feature of the pathological mechanism conferred by multiple clonal hematopoiesis driver genes, but it may also represent a component of a common feedforward mechanism by which multiple clonal hematopoiesis driver genes promote HSPC clonal expansion.

We acknowledge that this study has several limitations. First, while these data support the concept that macrophages have a major role in the *Ppm1d*-mediated phenotype, we cannot exclude the possibility that other immune cell populations also contribute to cardiac dysfunction in this model. However, it should be noted that myeloid, not lymphoid, cell infiltration of the myocardium was shown to predominate in the Ang II model of cardiac stress.^49^ Furthermore, as shown here, macrophages are the main source of the IL-1β cytokine. Second, since our experimental system involves myeloablation before BMT to allow for donor cell engraftment, we cannot exclude the potential confounding effect of irradiation on the cardiac resident immune cell populations in this model. Third, our system, employing CRISPR-Cas9 system, generates homozygous mutation with a relatively high VAF, and this could exaggerate the some of the phenotype observed over the short time course of this study. We note, however, that the murine models of clonal hematopoiesis/CVD are conducted over a short time span compared with the clinical condition. As with many murine models, a higher dose of stimulus is used to compensate for the abbreviated time course. Fourth, recent reports have documented that the CRISPR-Cas9 system can trigger the DDR in some cell types.^55^ However, previous studies have employed the CRISPR/Cas9 system to analyze the DDR pathway,^24^ and it has been reported that HSPC are relatively tolerant of CRISPR-induced dsDNA breaks, exhibiting rapid and transient DDR activation.^56^ In the current study, gene editing was performed ex vivo before BMT, and a month before the initiation of Ang II infusion. Furthermore, no cellular or cardiac phenotype could be detected when the CRISPR system was used to create dsDNA breaks using a sgRNA that targets a noncoding region of the gene that encodes for α-actin, and no DDR activation could be observed under baseline conditions in Cas9-expressing cells that were treated with the *Ppm1d*-targeting sgRNAs. Finally, aspects of these findings were corroborated by expressing the truncated PPM1D protein with an expression system that does not involve CRISPR. Collectively, there were no detectable effects from the CRISPR-Cas9 system in this clonal hematopoiesis model, consistent with reports that CRISPR-induced dsDNA breaks have little if any effect on HSPC function.^56^

## Conclusions

By employing CRISPR-Cas9 technology to introduce mutations in exon 6 of the *Ppm1d* gene in HSPC, we established a mouse model of t-CH that recapitulates many of the features observed in individuals with *PPM1D*-mediated clonal hematopoiesis. Mice transplanted with bone marrow containing *Ppm1d*-mutant cells were more susceptible to stress-induced cardiac remodeling and dysfunction. *Ppm1d*-mutant macrophages displayed DDR pathway suppression and greater cytokine production in response to stress, and NLRP3 inflammasome inhibition reversed the mouse phenotype that was conferred by the transplantation of the *Ppm1d*-mutant cells. These data suggest that t-CH, caused by the gain-of-function mutations in *PPM1D*, can contribute to the late cardiac toxicity observed in cancer survivors. If validated by further studies, these data suggest that observations of t-CH could be predictive of long-term cardiac dysfunction in cancer survivors, and that anti-inflammatory therapies could be useful in treating individuals with this condition.

## Author Contributions

Y. Yura, E. Miura-Yura, Y. Katanasaka, KD. Min, N. Chavkin, A. Polizio, H. Ogawa, K. Horitani, H. Doviak, M.A. Evans, M. Sano, Y. Wang, K. Boroviak, G. Philippos, A.F. Domingues, G. Vassiliou, and S. Sano conducted experiments and acquired and analyzed data. S. Sano provided advice for the overall study. K. Walsh supervised the study. Y. Yura and K. Walsh wrote the article. K. Walsh and S. Sano provided funding.

## Sources of Funding

This work was funded by National Institutes of Health grants HL131006, HL132564, and HL138014 to K. Walsh, HL152174 to S. Sano and K. Walsh, T32 HL007284 to N.W. Chavkin, and American Heart Association grant 20POST35210098 to M.A. Evans.

## Disclosures

None.

## Supplemental Materials

Expanded Methods

Data Supplement Figures I–XIX

Data Supplement Tables I–III

## Supplementary Material



## Novelty and Significance

### What Is Known?

- Patients who receive cancer therapy frequently develop short- and long-term cardiac dysfunction.
- Patients with cancer often exhibit therapy-related clonal hematopoiesis (t-CH), an aggressive form of clonal hematopoiesis.
- Whether t-CH contributes to development of cardiac dysfunction in cancer survivors is unknown.
