# Supplementary figures and images for: The Cancer Therapy-Related Clonal Hematopoiesis Driver Gene Ppm1d Promotes Inflammation and Non-Ischemic Heart Failure in Mice
Source: Circ Res. 2021 Jul 28;129(6):684–98. doi: 10.1161/CIRCRESAHA.121.319314 (PMC8409899; doi:10.1161/CIRCRESAHA.121.319314)

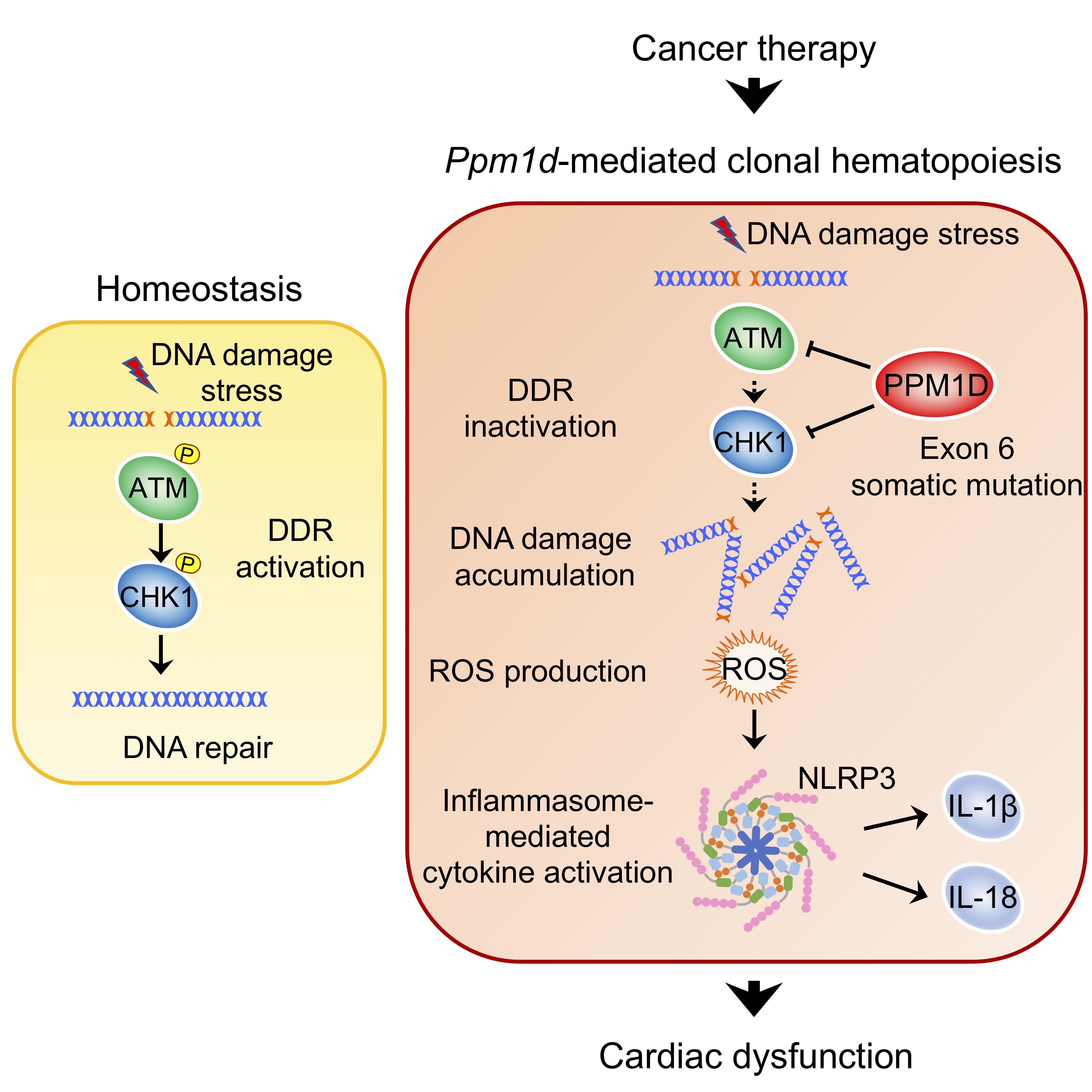

Supplement: Supplementary file 1 [file res-129-684-s001.jpg]
