# Supplementary material for: The Cancer Therapy-Related Clonal Hematopoiesis Driver Gene Ppm1d Promotes Inflammation and Non-Ischemic Heart Failure in Mice
Source: Circ Res. 2021 Jul 28;129(6):684–98. doi: 10.1161/CIRCRESAHA.121.319314 (PMC8409899; doi:10.1161/CIRCRESAHA.121.319314)
Supplement: Supplementary file 2 [file res-129-684-s002.pdf]

SUPPLEMENTAL MATERIAL

Supplemental Methods

Cell culture

HEK 293T and J774.1 cells were obtained from the American Type Culture Collection. Cells were cultured in DMEM supplemented with 10% FBS and penicillin/streptomycin/L-glutamine (Complete Media) at 37 °C under 5% CO2 in a humidified chamber. A sgRNA targeting exon 6 of *Ppm1d* gene was designed using CRISPR design tool. (https://zlab.bio/guide-design-resources) sgRNA1: gtcccagctgagatagctag. sgRNA2: tggcttaagtcgaagtagcg. CRISPR/Cas9-mediated gene editing technology was employed to establish PPM1D mutated J774.1 cells. EGFP-positive cells were sorted with an FACSAria (BD Bioscience) after 7 days incubation, and cells were used for experiments after expansion. Bone marrow-derived macrophages (BMDM) were isolated and cultured in Complete Media (RPMI1640 medium with 10% Fetal Bovine Serum) (Cat# 11-875-093 and Cat# SH3091003, respectively, Fisher Scientific). Bone marrow was flushed from the tibia and femurs of 1 month after BMT. Cells were washed and cultured overnight in Complete Media in the presence of Macrophage Colony Stimulating Factor (M-CSF) (100 ng/mL) (Cat#315-02, Peprotech). Differential plating was used to purify monocytes, that were identified as those cells that did not attach tissue culture plastic following 16 hours of culture. Macrophage proliferation and differentiation was induced by 2 days of culture in Complete Media in the presence of M-CSF. Cells were differentiated for 5 days and were detached by scraping. J774.1 cells and BMDM were stimulated with LPS for 6 hours in the presence or absence of 3.0 µM of PPM1D inhibitor GSK2830371 (Cat#5140, Tocris) or 10mM of 4-Hydroxy-TEMPOL (Cat#176141, Sigma Aldrich).

Lentivirus production

Lentivirus particles were generated as described.[29] Briefly, the plasmids (lentivirus vector, psPAX2 and pMD2.G) were co-transfected to HEK293 T cells using polyethyleneimine and the supernatant was collected 48 hours after transfection. After filtration (0.45µm), virus particles were concentrated by ultracentrifugation at a speed of 72,100 g for 3 hours. The virus pellet was suspended in Stemspan medium (Cat#09600, STEMCELL Technologies) and kept at -80 °C. Lentivirus particle titer was determined using Lenti-X qRT-PCR Titration kit (Cat# 631235, Clontech).

Isolation of lineage-negative bone marrow cells and lentivirus transduction

Lineage-negative bone marrow cells were isolated from B6(C)-Gt(ROSA)26Sorem1.1(CAG-cas9*,-EGFP)Rsky/J mice using the Lineage depletion kit (Cat# 130-090-858, Miltenyi Biotech). Cells were pre-incubated with the StemSpan medium (Cat# 09600, STEMCELL Technologies) for 1.5 hours at 37 °C. Lentivirus transduction was performed in the presence of 20ng/mL of Thrombopoietin (TPO) (Cat#315-14, PeproTech), 50ng/mL of Stem cell factor-1 (SCF-1) (Cat#250-03, PeproTech) and 4µg/mL of polybrene for 16-20 hours. Cells were collected and re-suspended with RPMI medium before transplantation.

Bone marrow transplantation

Recipient mice were exposed to two radiation doses of 5.5 Gy at 4 hours apart using RS 2000 Biological System irradiator (Rad Source, USA). For lineage-negative cell transplantation, lentivirus-transduced cells (5×10^5 cells in 200µl of RPMI1640 medium /mouse) were retro-orbitally injected into 8- to 10-week-old C57BL/6 wild type mice. Experimental mice were randomly assigned to each experimental or control group. We did not exclude any mice with the exception of mice that were not used due to human error during the experiment. While statistical analyses were not used to predetermine sample sizes, estimates of sample sizes to obtain statistically significant results were made based on our previous experimental findings with these models.

qRT-PCR

Total RNA from tissues and cultured cells was isolated using QIAzol reagent (Cat# 79306, QIAGEN) and NucleoSpin RNA Plus kit (Cat# 740984.50, Takara). RNA (0.5-1.2 µg) was reverse transcribed with QuantiTect Reverse Transcription Kit (Cat# 205313, QIAGEN). qRT-PCR was performed with Power SYBR® Green reagent (Cat# 4368708, ThermoFisher Scientific) in a ViiA7 PCR system. A standard thermocycling protocol (95 °C for 15 seconds and 60°C for 60 seconds, total 40 cycles) was used to amplify gene copy number. Primers for mouse gene expression studies are shown in the Supplemental Table II. Results were analyzed with the ΔΔCt method. 36b4 was used as the reference gene for normalization.

Hematopoietic cell parameter and flow cytometry analysis of peripheral blood and heart immune cells

Hematopoietic parameters were analyzed using the Element HT5 Veterinary Hematology Analyzer (Heska). Flow cytometric analysis of peripheral blood leukocytes and cardiac immune cells was performed at the time points indicated as described previously.[19] The antibodies used for flow cytometric analysis are listed in Supplemental Table III. Peripheral blood cells were obtained from the retro-orbital vein and collected into K2 EDTA-containing BD microtainer blood collection tubes (Cat# 365974, BD Biosciences). Red blood cells were lysed with eBioscience 1X RBC Lysis Buffer (Cat# 00-4333-57, Thermo Fisher Scientific) for 5 minutes on ice. Incubation with antibodies was performed for 20 minutes at room temperature in the dark. To analyze cardiac immune cells, the right atrium was removed, and hearts were flushed with 15 ml of cold phosphate-buffered saline (PBS) from the apex. Left ventricles were minced and digested in collagenase I (450 U/ml), collagenase XI (125 U/ml), hyaluronidase (450 UI/ml), and DNase I (60 U/ml) (Cat# C0130, C7657, H3506, and D4513, respectively, MilliporeSigma) at 37°C for 30 minutes using a ThermoMixer C (Eppendorf) at 900 rpm. Hearts were subsequently homogenized through cell strainers (Falcon, Cat# 352350, Thermo Fisher Scientific). After incubation with antibodies, dead cells were excluded from analysis by Zombie Aqua or Zombie violet (Cat# 423102 and Cat# 423113, respectively, BioLegend) staining according to the manufacturer's instructions. For determining cell numbers, 123count eBeads (Cat# 01-1234-42, Thermo Fisher Scientific) were used. A Fortessa was used for data acquisition. Data were analyzed with FlowJo software (Tree Star, Inc.) and cell numbers were normalized as number of cells/100 mg wet weight of the heart. Cells were defined as described in the gating strategy shown in the relevant Supplemental Figures.

Echocardiographic analyses

Transthoracic echocardiography was performed on isoflurane-anesthetized mice. Mice were kept semi-awake in a shallow anesthesia state by monitoring responses to physical stimuli (tail pinch, etc.), and heart rate was maintained at approximately 500-600 bpm. The left ventricle end-systolic diameter (LVDs), LV end-diastolic diameter (LVDd), end-diastolic interventricular septum (IVSd), and LV end-

diastolic posterior wall thickness (LVPWd) were measured in the short-axis M-mode view at the level of the mid papillary muscles with a Vevo 1100 imaging system (FUJIFILM VisualSonics, Inc, Toronto, ON, Canada) equipped with an MS400 (18-38 MHz) phased-array transducer.

Pump implantation

To induce cardiac dysfunction in mice, osmotic minipumps (Alzet model 2004; Cupertino, CA) containing either angiotensin II (AngII; 2.0 mg/kg/day, diluted in sterile saline, Sigma-Aldrich) or saline (Sham) were implanted subcutaneously into a small pocket made through an incision at the nape neck. Mice were anesthetized with isoflurane during the entire surgical procedure and the wounds were closed with wound clips. Osmotic minipumps were primed in PBS at 37°C for 24 hours before implantation, and they remained in place for 28 days post-implantation. In some experiments, mice were implanted with a second minipump to prolong the time course. For the inflammasome inhibition experiment, osmotic minipumps containing MCC950 (5 mg/kg/day, diluted in sterile PBS, Cat# S7809, Selleck Chemicals) or PBS, were implanted at the same time as the AngII-containing pumps. Blood pressure was measured by tail cuff plethysmography, as previously described.

BNP

Plasma brain natriuretic peptide was quantified by the BNP Enzyme Immunoassay Kit according to the manufacturer’s instructions (Cat# EIAM-BNP-1, RayBiotech Inc., Norcross, GA).

ELISA

IL-1β and IL-18 were analyzed in bone marrow derived macrophage culture supernatants using commercial enzyme-linked immunoabsorbent assays (ELISA) according to manufacturer’s instructions (Cat# MLB00C, Cat#7625, R&D Systems).

DCF assay

Reactive Oxygen Species (ROS) was analyzed by 2', 7'–dichlorofluorescein (DCF) assay in J774.1 cell using commercial Cellular ROS Assay Kit (Cat#ab113851, Abcam.) according to manufacturer's instructions.

Histology

Heart tissues was perfused with cold PBS from the apex and fixed in 10% formalin at 4 °C overnight. Samples were processed for paraffin embedding, and 7-μm-thick sections were cut. In order to stain the tissue with the relevant stain, sections were deparaffinized and rehydrated. To determine the cardiomyocyte cross sectional area (CSA), heart sections were stained with Alexa Fluor 488 conjugated wheat germ agglutinin (Cat# W11261, Life Technologies) for 1 hour at room temperature. An observer who was blinded to the identities of the experimental groups quantified cardiomyocyte cross-sectional analysis (CSA) by computer assisted morphometric analysis of microscopy images acquired on a Keyence BZ-X710 microscope. The CSA of more than 500 randomly selected, cardiomyocytes per each group were used for analysis. For Picrosirius red/Fast Green staining, sections were incubated with freshly prepared staining buffer (1.2%/w saturated picric acid in water, 0.1%/w Fast Green FCF and 0.1%/w Direct Red 80) for at least 1 hour at room temperature (Cat# 197378, Cat# F7252, Cat# 365548, respectively, Sigma-Aldrich). Sections were washed briefly in acidified water (0.1%/v glacial acetic acid in water) and then dehydrated. Slides were mounted with coverslips using permanent mounting medium (Cat# H-5000, Vector Laboratories). The images were analyzed using Image J software (NIH) for quantification of fibrosis.

Western Immunoblotting

Bone marrow-derived macrophages and neutrophils isolated from mice, and J774.1 cells, were lysed with 50 mmol/L Tris·HCl, 150 mmol/L NaCl, 10 mmol/L EDTA, 1% Triton and Protease Inhibitor Cocktail (Cat# PI78440, Thermo Scientific). The protein concentration was measured with Pierce™ BCA Protein Assay Kit (Cat# 23227, Thermo Scientific). Samples were incubated with Blue Loading Buffer (Cat# B7703S, BioLabs) with 40 mM DTT for 5 min at 95 °C. Samples containing equal

amounts of protein were separated by SDS/PAGE (Cat# 4561033, Bio-Rad). After blocking with 5% skim milk in PBS with Tween 20 (0.1%) for 1 hour, the membranes were incubated with the indicated antibodies overnight at 4 °C, followed by HRP-conjugated second antibody (Cat# sc-2357, Santa Cruz Biotechnology) 1 hour at room temperature. The following antibodies were used for immunoblotting: rabbit anti-phospho-Atm (Ser1981) (D25E5), Chk1 (Ser345), Histone H2A.X (Ser139) (20E3), p38 (Thr180/Tyr182), p65 (Ser536) (93H1), WIP1 (D4F7), and GAPDH (14C10) (Cat# 13050, #2341, #9718, #9211, #3033, #11901, #2118, respectively, Cell Signaling Technology). Images were visualized using ECL™ Prime Western Blotting System (Cat# RPN2232, GE Healthcare). Relative protein levels were quantified using ImageJ (National Institutes of Health, Bethesda, MD, USA).

Comet assay

DNA-damage was quantified with the 3-well slide Comet Assay Kit (Cat# ab238544, Abcam. Fluorescent images were taken by a Keyence BZ-X710 microscope. The images were analyzed using Image J software (NIH). Tail DNA percentage and Olive Tail Moment were calculated as 100 x Tail DNA Intensity/Cell DNA Intensity and Tail DNA% x Tail Moment Length, respectively.

Immunostaining

For IL-1β staining, cardiac tissue sections were deparaffinized and antigen retrieval was performed using citric acid buffer (Cat# H-3300, Vector Laboratories, Inc.). Sections were then blocked with 2.5 % goat serum for 1 hour. Subsequently, cardiac tissue sections were incubated with primary antibody specific for IL-1β (polyclonal rabbit IgG, Cat# BS-6319R, Bioss), and Mac3 (rat IgG, clone M3/84, Cat# sc-19991, Santa Cruz Biotechnology) for overnight at 4°C. IL-1β was visualized with biotinylated anti-rabbit IgG following fluorescein labelled streptavidin (Vector Laboratories) and Mac3 with Alexa Flour 594-conjugated anti-rat IgG (Cat# A-21209, Life Technologies). To distinguish *bona fide* target staining from the background, secondary antibody only was used as a negative control in each experiment. Nuclei were stained with DAPI, and slides were incubated with Sudan Black B (Cat# 3545, Sigma-Aldrich) to reduce autofluorescence. Cell death was determined using the terminal deoxynucleotidyl transferase dUTP nick-end labelling (TUNEL) method using In Situ Cell Death

Detection Kit (Cat# 11684795910, Roche) according to manufacturer's instructions. Fluorescent images were taken by a BZ-9000 Keyence microscope.

Cell sorting

For qPCR analysis of the cardiac macrophage ($CD45^{+}Ly6G^{-}CD64^{+}Ly6C^{-}$), myeloid cell ($CD45^{+}CD31^{-}CD11b^{+}$), fibroblast ($CD45^{-}Ter119^{-}CD31^{-}PDGFR\alpha^{+}Sca1^{+}$), endothelial cells ($CD45^{-}CD11b^{-}CD31^{+}Ter119^{-}$), heart digests were prepared as described above and sorting was performed on Influx Cell Sorter (BD Biosciences) platform with a 100 μM nozzle and flow pressure set to 20 psi. A total of 10,000 cells were sorted for each population. RNA was extracted using RNeasy extraction kit (#Cat74004, Qiagen).

Supplemental Figures

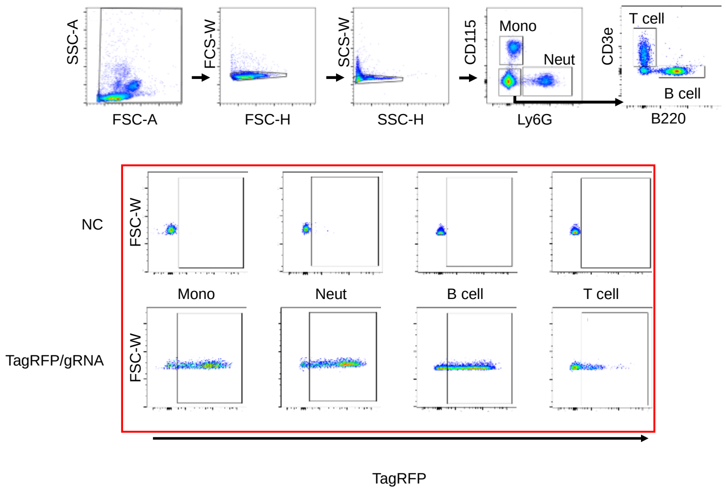

Supplemental Figure I. Flow cytometry gating strategy for analysis of peripheral blood cells. Cells are defined as: Monocytes (Mono; CD115^{+}), Neutrophils (Neut; Ly6G^{+}), B cells (CD115^{-}Ly6G^{-}B220^{+}), T cells (CD115^{-}Ly6G^{-}CD3e^{+}). Lentivirus-transduced cells encoding sgRNA are TagRFP-positive. NC = negative control.

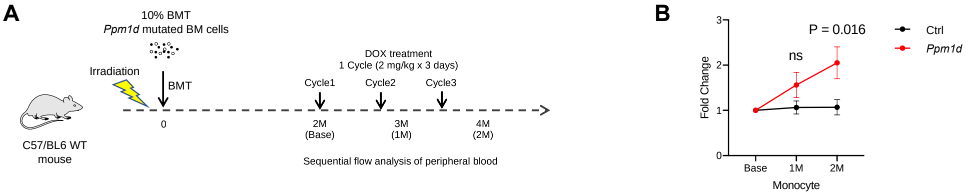

Supplemental Figure II. A. Experimental design of competitive bone marrow transplantation (BMT) experiments. Bone marrow chimaeras in lethally irradiated C57/BL6 wild-type mice were generated by the competitive transplantation of Cas9 bone marrow cells that were transduced with TagRFP-tagged, lentiviral vectors encoding a sgRNA targeting exon 6 of *Ppm1d* or a control sgRNA. Transduced bone marrow cells were mixed with Cas9 bone marrow cells that were transduced with EGFP-expressing lentivirus vector at a 1:9 ratio, respectively. Doxorubicin (DOX) treatment (one cycle; 2 mg/g x 3 days, interval 21 days) was initiated at 2 months (M) post-BMT, and peripheral blood was collected for flow cytometric analysis at 1 M and 2 M after the initiation of DOX administration. **B**. Flow cytometric analysis of peripheral blood monocytes in mice exposed to DOX treatment. Cells derived from HSPC with PPM1D or control sgRNAs were identified through detection of their fluorophores (n=5 for Ctrl and n=7 for *Ppm1d* group). Statistical significance was evaluated by two-way repeated ANOVA with Sidak multiple comparison tests.

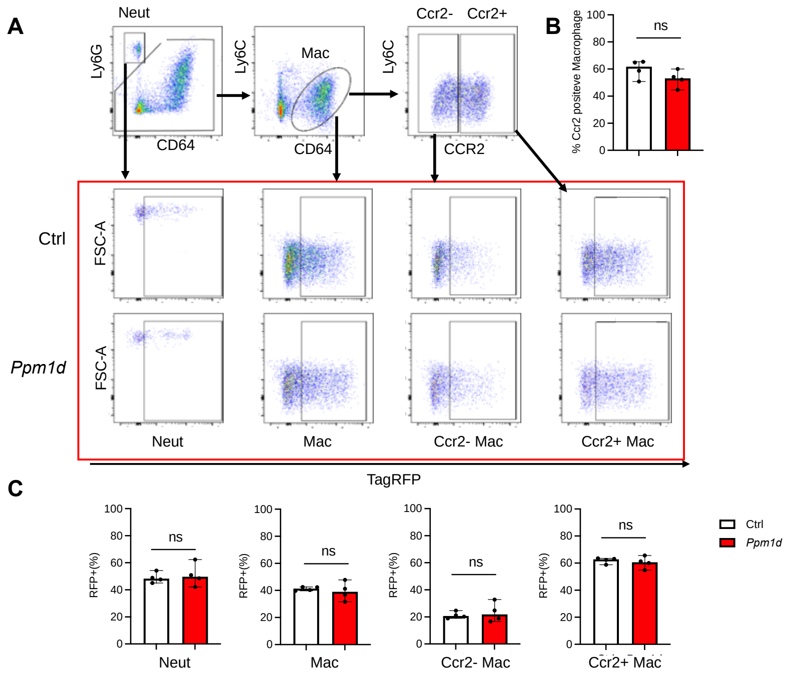

Supplemental Figure III. **A**. Analysis of cardiac immune cells by flow cytometry in sham mice that had been transplanted with HSPCs transduced with control (Ctrl, non-targeted) sgRNA or a sgRNA that targets exon 6 of the *Ppm1d* gene. Cell populations analyzed were neutrophil (Neut; CD45$^+$CD64$^-$Ly6G$^+$), macrophages (Mac; CD45$^+$Ly6G$^-$CD64$^+$Ly6C^{lo}), Ccr2- macrophages (Ccr2- Mac; CD45$^+$Ly6G$^-$CD64$^+$Ly6C^{lo}Ccr2-) and Ccr2+ macrophages (Ccr2+ Mac; CD45$^+$Ly6G$^-$CD64$^+$Ly6C^{lo}Ccr2+). **B**. Quantitation of Ccr2-positive macrophage population in experimental groups 4-weeks after bone marrow transplant (n=4 per genotype). Statistical significance was evaluated by Mann Whitney U tests. **C**. Quantitation of % of TagRFP-positive fractions of cardiac neutrophil, macrophages, Ccr2-negative macrophages, and Ccr2-positive macrophages (n=4 per genotype). Statistical significance was evaluated by Mann Whitney U tests.

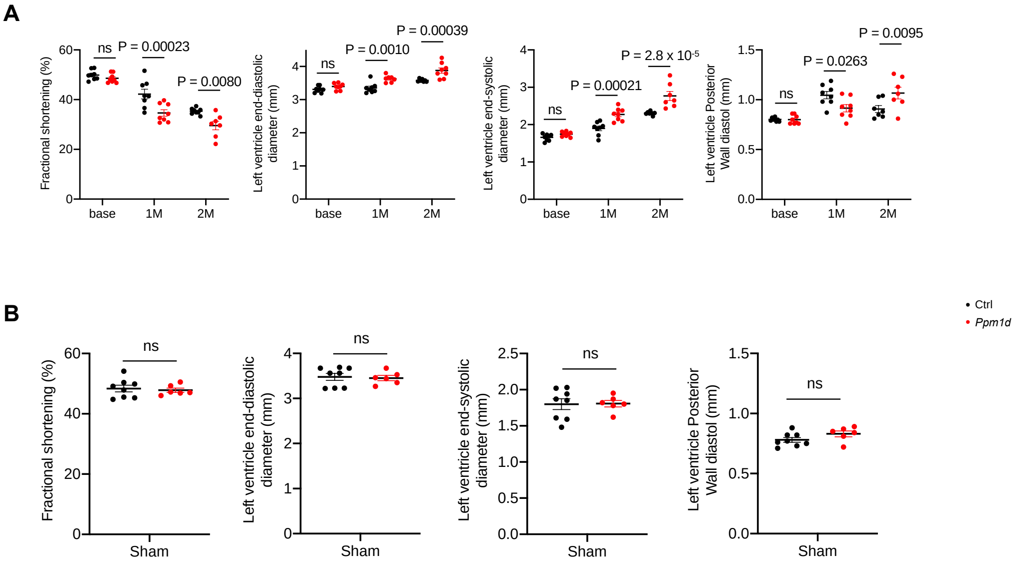

Supplemental Figure IV. **A.** Prolonged sequential echocardiographic analysis of mice transplanted with HSPC transduced with lentiviral vectors encoding control (Ctrl) or *Ppm1d*-targeting (n=8 per genotype) at baseline (Base), 1 month (1M), and 2 months (2M) after AngII infusion. Statistical significance was evaluated by a two-way repeated ANOVA with Sidak multiple comparison tests. **B.** Echocardiographic analysis of mice transplanted with HSPC transduced with lentiviral vectors encoding control (Ctrl) or *Ppm1d*-targeting (n=8 and 6 respectively) at sham condition. Statistical significance was evaluated by two-tailed unpaired Student's t test.

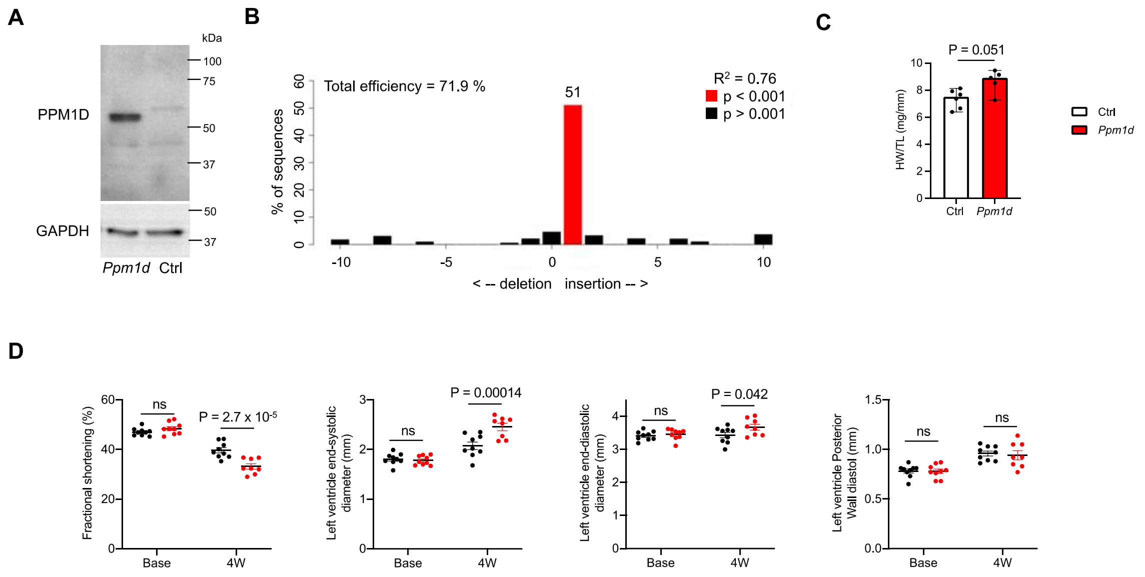

Supplemental Figure V. Corroboration of greater AngII-induced cardiac remodeling in mice harboring HSPC treated with a second, independent sgRNA that targets exon 6 of *Ppm1d*. **A**. PPM1D expression assessed by immunoblot analysis in bone marrow derived macrophages. **B**. TIDE analysis reveals the nature of the insertions and deletions. **C**. Heart weight (HW) adjusted by tibia length (TL) following 4 weeks AngII infusion for both experimental groups. (n=6 for Ctrl and n=5 for *Ppm1d* group). Statistical significance was evaluated Mann Whitney U tests. **D**. Sequential echocardiographic analysis of mice transplanted with HSPC transduced with lentiviral vectors encoding control (Ctrl) or *Ppm1d*-targeting (n=6 per genotype) at baseline (Base) and 4 weeks (W) after AngII infusion. Statistical significance was evaluated by a two-way repeated ANOVA with Sidak multiple comparison tests.

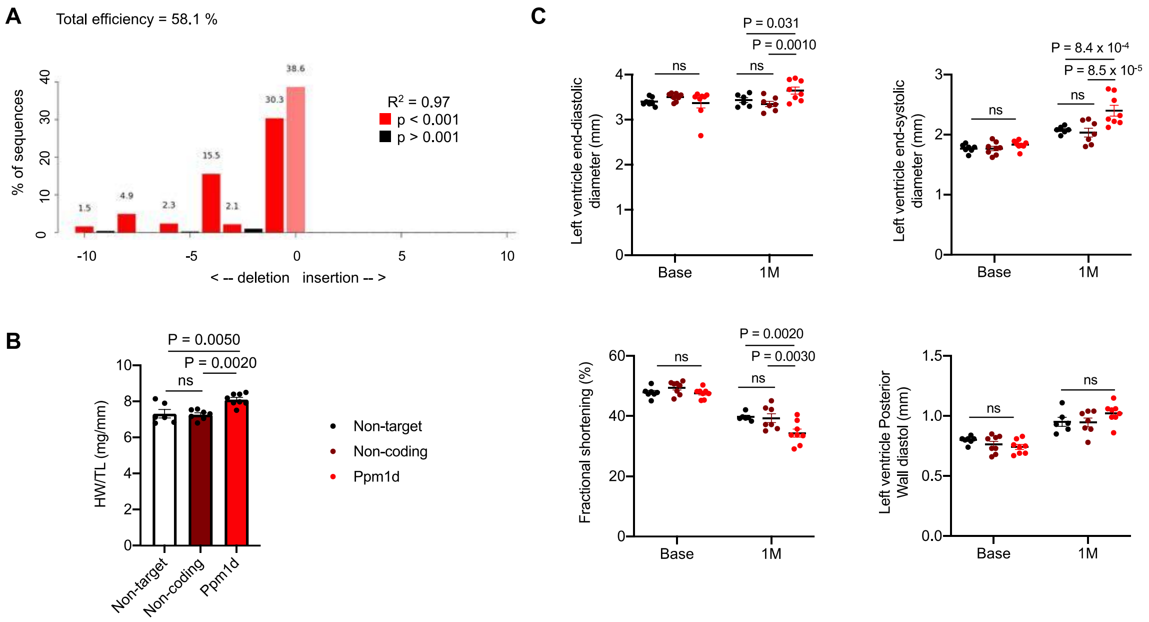

Supplemental Figure VI. Corroboration of greater AngII-induced cardiac remodeling in mice harboring HSPC treated with a sgRNA that targets exon 6 of *Ppm1d* compared with a control sgRNA that target the intronic region of the gene encoding α-actin **A.** TIDE analysis to show the nature of insertions and deletion in the non-coding (intronic) region of the mouse gene that encodes α-actin. **B.** Heart weight (HW) adjusted by tibia length (TL) following 28 days AngII infusion for mice transplanted with HSPC transduced with lentiviral vectors encoding 2 controls (Non-target and Non-coding) or *Ppm1d*-targeting. (n=6 for Non-target, n=7 for Non-coding, and n=8 for *Ppm1d* group). Statistical significance was evaluated by one-way ANOVA with a Tukey multiple-comparison test. **C.** Sequential echocardiographic analysis of 3 experimental groups (n=6 for Non-target, n=7 for Non-coding, and n=8 for *Ppm1d* group) at baseline (Base) and 1 month (1M) after AngII infusion. Statistical significance was evaluated by a two-way repeated ANOVA with Sidak multiple comparison tests.

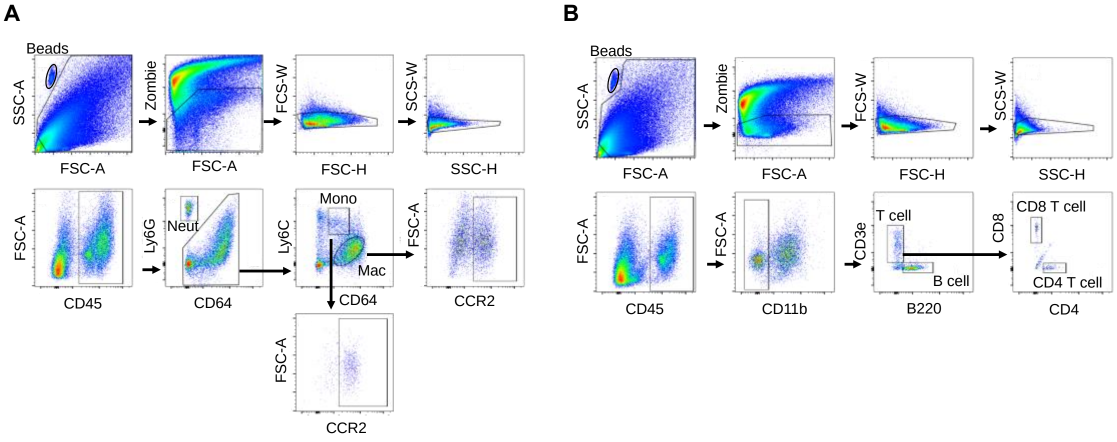

Supplemental Figure VII. A. Flow cytometry gating strategy for cardiac immune cells. Cells are defined as: Neutrophils (Neut; $CD45^{+}Ly6G^{+}$), $Ly6C^{hi}$ monocytes ($Ly6C^{hi}$mono; $CD45^{+}Ly6G^{-}CD64^{int}Ly6C^{hi}$), macrophages (Mac; $CD45^{+}$Ly6G-$CD64^{+}Ly6C^{lo}$). **B**. Cells are defined as: CD4 T cells ($CD45^{+}CD11b^{-}CD3e^{+}CD4^{+}$), CD8 T cells ($CD45^{+}CD11b^{-}CD3e^{+}CD8^{+}$), B cells ($CD45^{+}CD11b^{-}CD3e^{-}B220^{+}$).

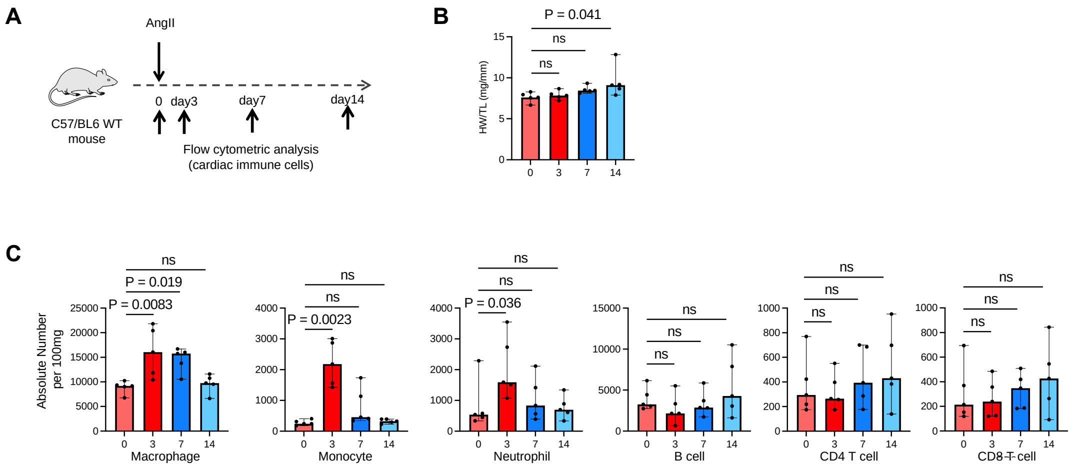

Supplemental Figure VIII. A. Schematic of experimental design. C57/BL6 WT mice were infused with AngII (2.0mg/kg/day) and cardiac immune cell numbers were analyzed by flow cytometry at 0, 3, 7, 14 days. **B**. Heart weight (HW) adjusted by tibia length (TL) at 0, 3, 7, 14 days after AngII infusion (n=5 per timepoint). Statistical significance was evaluated by Kruskal-Wallis tests with Dunn's multiple comparison tests. **C**. Quantitation of flow cytometry analysis of immune cells in the heart at 0, 3, 7, 14 days after AngII infusion (n=5 per timepoint). The data are shown as absolute number per 100 mg. Statistical significance was evaluated by Kruskal-Wallis tests with Dunn's multiple comparison tests.

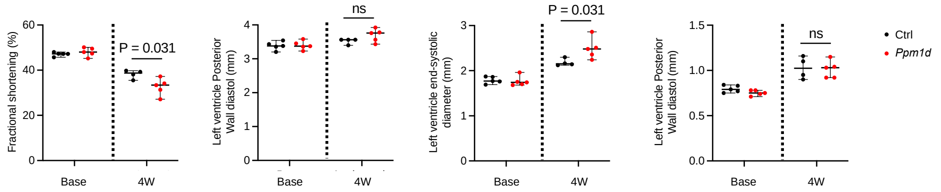

Supplemental Figure IX. Sequential echocardiographic analysis of *Ccr2*-KO mice transplanted with HSPC transduced with lentiviral vectors encoding control (Ctrl) or *Ppm1d*-targeting (n=5 per genotype) at 0 (Base) and 4 weeks (W) post-AngII infusion. Statistical significance was evaluated by Mann Whitney U tests at the 4 week time point.

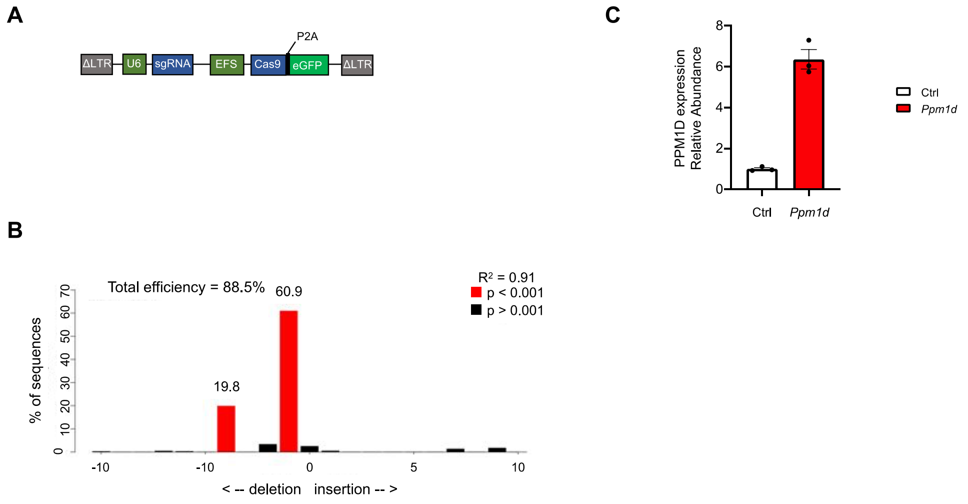

Supplemental Figure X. CRISPR gene editing in J774.1 cells**. A.** Depiction of the lentiviral vector containing the sgRNA, targeting *Ppm1d*, under the control of the U6 promoter (U6) and Cas9 under the control of the short EF1a promoter (EFS). EGFP is expressed in a bi-cistronic manner using picornavirus-derived 2A auto-cleavage site (P2A) system. **B.** TIDE analysis reveals the nature of the insertions and deletions. **C.** Quantification of PPM1D protein expression in J774.1 cells for control (Ctrl) and *Ppm1d*-targetted groups. (n=3 per genotype).

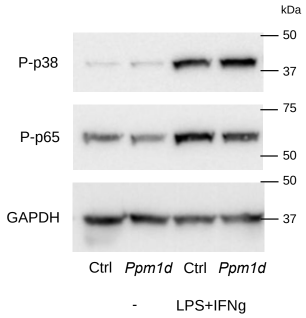

Supplemental Figure XI. P38 and p65 phosphorylation (P) and GAPDH protein expression evaluated by immunoblot analysis in J774.1 cells treated with lentivirus with control (Ctrl) or Ppm1d-tartgetted sgRNA. The cells were stimulated with 20 ng/ml LPS/ 50ng/ml IFN-γ for 6 hours.

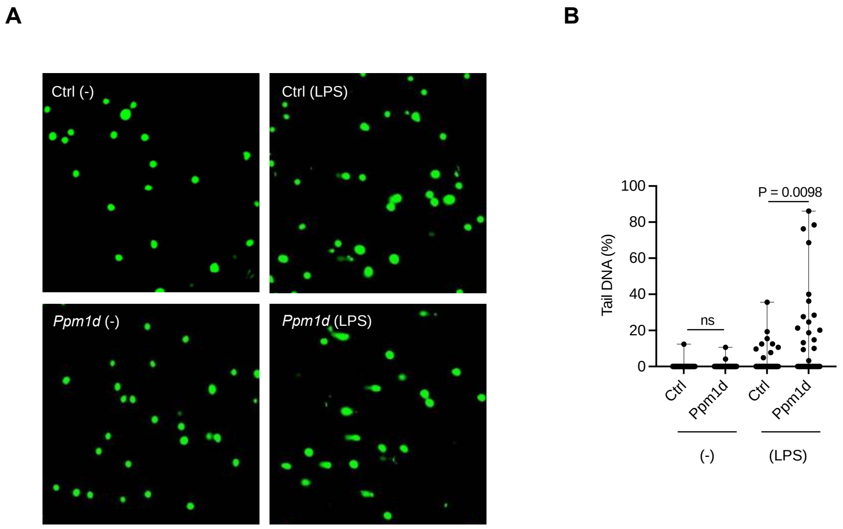

Supplemental Figure XII. Comet assay analysis of bone marrow-derived macrophages from control and *Ppm1d* gene-edited mice. **A.** Representative images from the comet assay comparing control (Ctrl) and *Ppm1d*-targetted groups in the presence and absence of LPS. Representative images were selected to represent the mean value of each condition. **B.** Quantitative analysis of the comet assay for the different experimental groups by assessing the Tail DNA (%) (n = 47, 48, 48, 45 for each group, respectively). Statistical significance was evaluated by Kruskal-Wallis tests with post hoc Dunn multiple comparison tests.

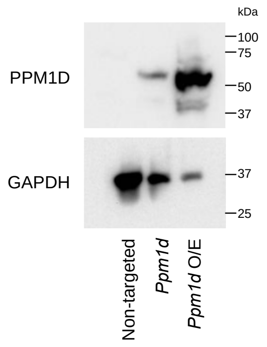

Supplemental Figure XIII. Western blot analysis of PPM1D protein expression in J774.1 cells treated with a control (non-targeted) sgRNA, cells with *Ppm1d* gene editing, and cells that overexpress (O/E) the truncated form of murine *Ppm1d* from a lentivirus vector.

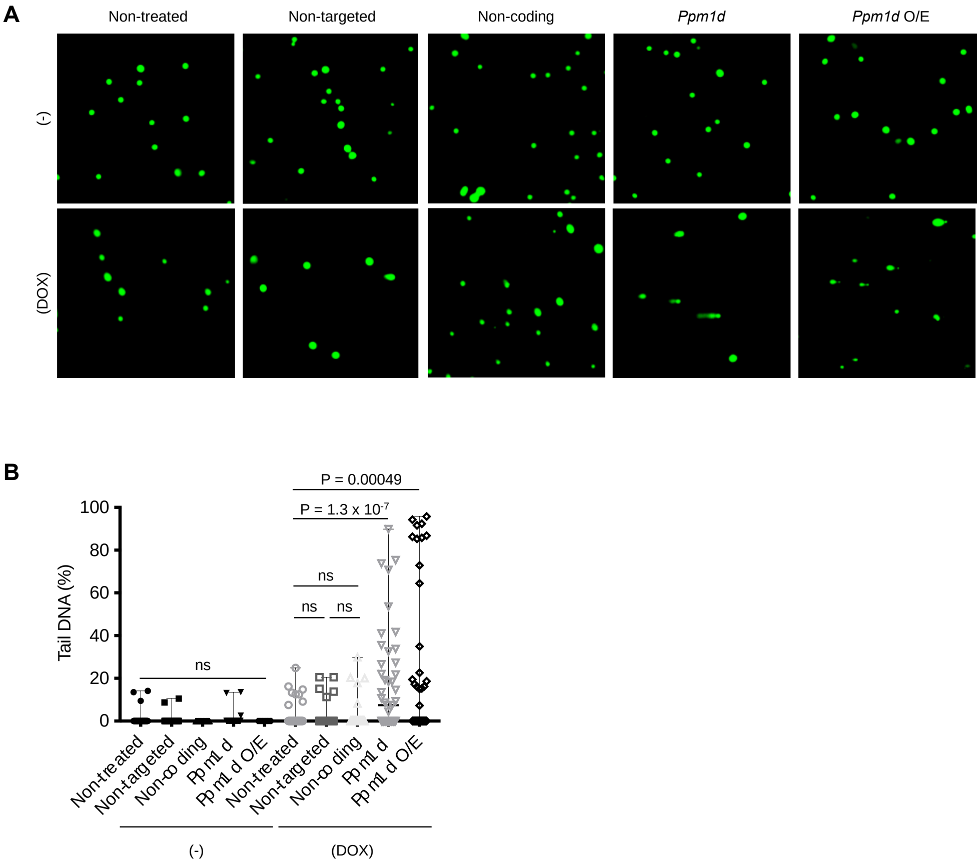

Supplemental Figure XIV. A. Representative images from the comet assay in J774.1 cells treated with or without presence or absence of doxorubicin (DOX). Cell conditions are non-treated, treated with a Cas9/sgRNA that is not targeted to DNA (non-targeted), Cas9/sgRNA that targets the intron of α-actin (non-coding), Cas9/sgRNA that targets exon 6 of *Ppm1d* gene (*Ppm1d*) and lentivirus-mediated overexpression of the truncated form *Ppm1d* (*Ppm1d* O/E). Representative images were selected to represent the mean value of each condition. **B.** Quantitative analysis of the comet assay for the different experimental groups by assessing the tail DNA (%) (n = 53, 45, 54, 48, 55, 57, 52, 56, 47, 49 for each group, respectively). Statistical significance was evaluated by Kruskal-Wallis tests with post hoc Dunn multiple comparison tests.

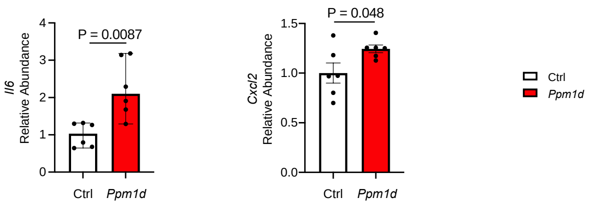

Supplemental Figure XV. Gene expression analysis of control (Ctrl) and *Ppm1d*-mutated J774.1 cells at 6 h after stimulation with 10 ng/mL lipopolysaccharide (LPS) (n=6 per group). Statistical significance was evaluated by Mann Whitney U tests for *Il6* and two-tailed unpaired Student's t test for *Cxcl2*.

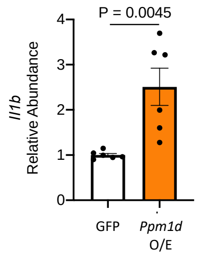

Supplemental Figure XVI. *Il1b* transcript expression analysis in J774.1 cells expressing GFP or PPM1D truncation form (*Ppm1d* O/E) at 6 hours after stimulation with 10 ng/mL LPS (n=6 per group). Statistical significance was evaluated by two-tailed unpaired Student's t test.

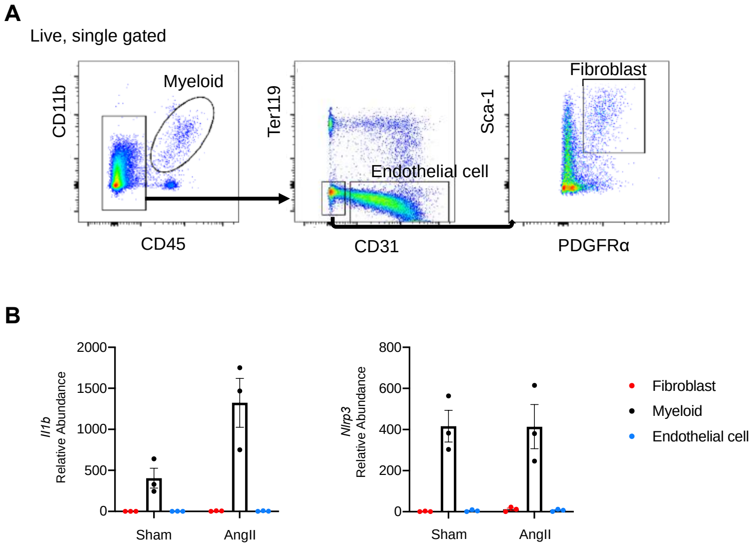

Supplemental Figure XVII. Expression of *Il1b* and *Nlrp3* is largely confined to cardiac macrophages. **A**. Flow cytometry gating strategy for cardiac myeloid cells, endothelial cells, and fibroblasts. Cells are defined as: fibroblast (CD45^{-}Ter119^{-}CD31^{-}PDGFRα^{+}Sca1^{+}), myeloid cells (CD45^{+}CD11b^{+}), and endothelial cell (CD45^{-}CD11b^{-}CD31^{+}Ter119^{-}). These cells were sorted in both sham hearts and 3 days of AngII treatment. **B**. Gene expression analysis of *Il1b* and *Nlrp3* in these 3 populations both in sham and after 3 days of AngII stimulation (n=3 per group).

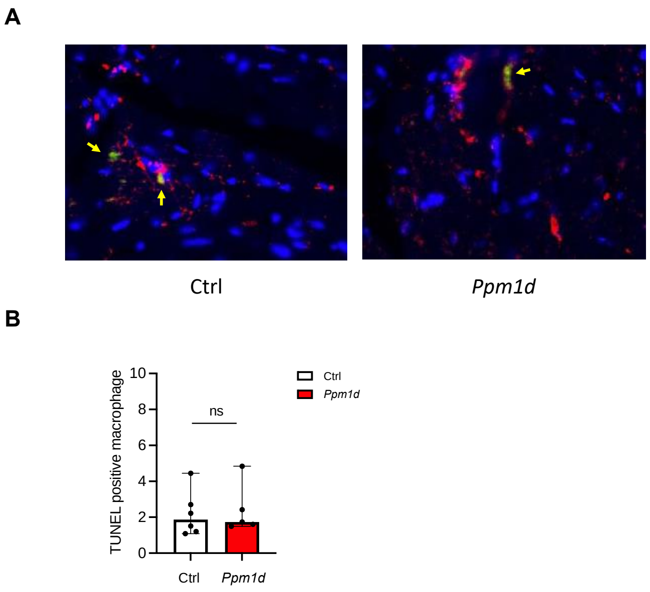

Supplemental Figure XVIII. A. Representative images of TUNEL staining (green) in Mac3-positive macrophages (red) from control (Ctrl) and *Ppm1d*-mutated mice. Representative images were selected to represent the mean value of each condition. **B**. Quantification of TUNEL positive macrophages in heart tissues at the termination of the experiment. (n=6 for Ctrl and n=5 for *Ppm1d* group). Statistical significance was evaluated by Mann Whitney U tests.

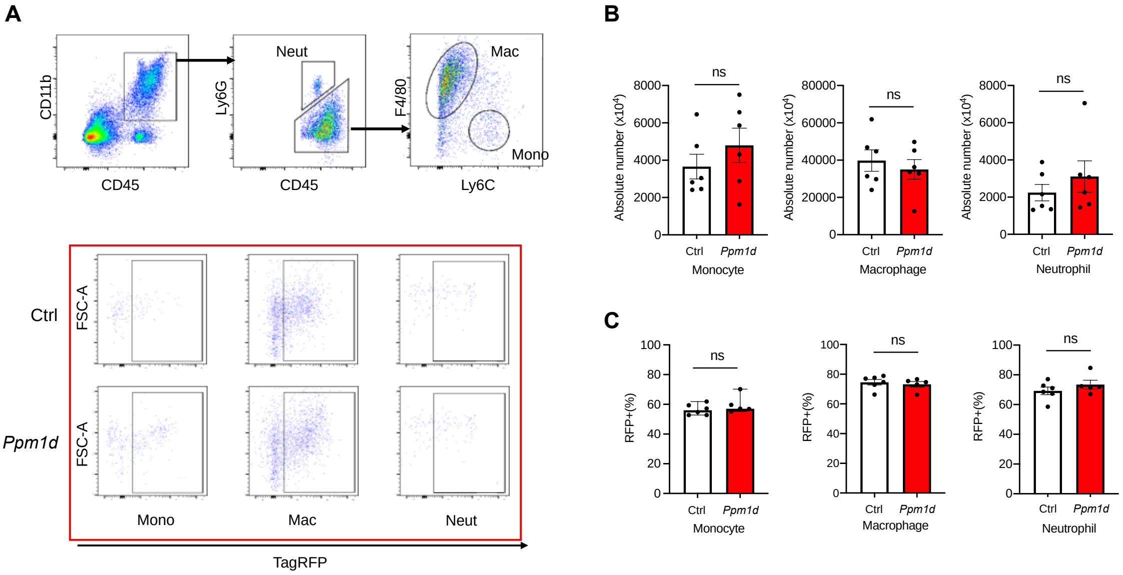

Supplemental Figure XIX. Heart tissue from AngII-treated mice transplanted with either WT or *Ppm1d*-mutant HSPCs did not differ in the absolute numbers of monocytes, macrophages or neutrophils within the heart between the two experimental groups. **A**. Analysis of cardiac immune cells by flow cytometry after 4 weeks of AngII infusion in mice that had been transplanted with HSPCs transduced with control (Ctrl, non-targeted) sgRNA or a sgRNA that targets exon 6 of the *Ppm1d* gene. Cell populations analyzed were neutrophils (Neut; $CD45^{+}CD11b^{+}Ly6G^{+}$), monocytes (Mono; $CD45^{+}CD11b^{+}Ly6G^{-}F4/80^{-}Ly6C^{hi}$) and macrophages (Mac; $CD45^{+}CD11b^{+}Ly6G^{-}F4/80^{+}Ly6C^{lo}$). **B**. Quantitation of major immune cell populations in experimental groups at the end of study. (n=6 per genotype). The data are shown as absolute number per 100 mg. Statistical significance was evaluated by two-tailed unpaired Student's t test. **C**. Quantitation of proportion of TagRFP-positive fractions of cardiac monocytes, macrophages and neutrophils revealed no differences between experimental groups at the end of study. (n=6 per genotype). Statistical significance was evaluated by Mann Whitney U tests for monocyte and two-tailed unpaired Student's t-tests for macrophage and neutrophil.

Supplemental Tables

Supplemental Table I. Basal heart function examined by echocardiography

	Ctrl	*Ppm1d*	
HR (bpm)	563.5 ± 13.5	553 ± 17.8	ns
LVDd (mm)	3.33 ± 0.05	3.31 ± 0.14	ns
LVDs (mm)	1.73 ± 0.07	1.73 ± 0.07	ns
FS (%)	48.1 ± 2.45	47.7 ± 1.49	ns
LVPWd (mm)	0.8 ± 0.04	0.77 ± 0.04	ns
IVSd (mm)	0.83 ± 0.03	0.81 ± 0.04	ns

Basal heart function examined by echocardiography at 4 weeks after BMT. (n=9 per genotype). HR indicates heart rate; LVDd, Left ventricle end-diastolic diameter; LVDs, Left ventricle end-systolic diameter; FS, Fractional shortening; LVPWd, Left ventricle posterior wall thickness at end diastole; IVSd, interventricular septum thickness at end diastole; ns, not significant. Statistical significance was evaluated by two-tailed unpaired Student’s t test.

Supplemental Table II. Primers used for quantitative PCR analysis

Gene Name	*Species*		
36b4	*Mus Musculus*	Forward	5'- GCTCCAAGCAGATGCAGCA -3'
		Reverse	5'- CCGGATGTGAGGCAGCAG -3'
IL18	*Mus Musculus*	Forward	5’- CAAACCTTCCAAATCACTTCCT -3'
		Reverse	5’- TCCTTGAAGTTGACGCAAGA -3'
Il1b	*Mus Musculus*	Forward	5'- TGACAGTGATGAGAATGACCTGTTC -3'
		Reverse	5'- TTGGAAGCAGCCCTTCATCT -3'
Il6	*Mus Musculus*	Forward	5'- GCTACCAAACTGGATATAATCAGGA -3'
		Reverse	5'- CCAGGTAGCTATGGTACTCCAGAA -3'
Nlrp3	*Mus Musculus*	Forward	5’- ATTACCCGCCCGAGAAAGG -3'
		Reverse	5’- TCGCAGCAAAGATCCACACAG -3'
Nppa	*Mus Musculus*	Forward	5’- AAGAACCTGCTAGACCACCTG -3’
		Reverse	5’- TGCTTCCTCAGTCTGCTCAC -3’
Nppb	*Mus Musculus*	Forward	5’- CTGAAGGTGCTGCCCCAGATG -3’
		Reverse	5’- GACGGATCCGATCCGGTC -3’

Supplemental Table III. Antibodies used for flow cytometry analysis.

Peripheral Blood				
Antibodies	Fluorescein	Clone	Source	Identifier
CD115	PE-Cy7	AFS98	Thermo Fisher	# 25-1152-82
Ly6C	APC	AL21	BD Biosciences	# 560595
Ly6G	PerCP5.5	1A8	BD Biosciences	# 560602
B220	APC-Cy7	RA3-6B2	BD Biosciences	# 552094
CD3	BV711	145-2C11	BioLegend	# 100349
CD4	FITC	RM4-5	Thermo Fisher	# 11-0042-82
CD8a	BV510	53.6-7	BioLegend	# 100751

Heart 1				
Antibodies	Fluorescein	Clone	Source	Identifier
CD45.2	PerCP5.5	104	BioLegend	# 109828
CD64	BV711	X54-5/7.1	BioLegend	# 139311
CCR2	BV421	SA203G11	BioLegend	# 150605
Ly6G	PE-Cy7	1A8	BioLegend	# 127618
Ly6C	FITC	HK1.4	BioLegend	# 128006
Live dead	Zombie aqua		BioLegend	# 423102

Heart 2				
Antibodies	Fluorescein	Clone	Source	Identifier
CD45.2	PerCP5.5	104	BioLegend	# 109828
CD11b	AF700	M1/70	BioLegend	#101222
CD3	PE-eFlour610	145-2C11	Thermo Fisher	# 61-0031-82
CD8a	BV510	1A8	BioLegend	# 100752
CD4	FITC	RM4-5	Thermo Fisher	# 11-0042-82
CD19	APC-Cy7	SJ25C1	BioLegend	# 363009
Live dead	Zombie violet		BioLegend	# 423113

Heart 3				
Antibodies	Fluorescein	Clone	Source	Identifier
CD45.2	PerCP5.5	104	BioLegend	# 109828
CD11b	APC-Cy7	M1-70	BioLegend	# 101226
Ly6G	PE	1A8	BioLegend	# 127602
Ly6C	APC	AL-21	BD Biosciences	# 560595
F4/80	PE-Cy7	BM8	BioLegend	# 123116
Live dead	Zombie aqua		BioLegend	# 423102

Heart 4				
Antibodies	Fluorescein	Clone	Source	Identifier
CD45.2	PerCP5.5	104	BioLegend	# 109828
CD64	APC	X54-5/7.1	BioLegend	# 139306
CCR2	BV421	SA203G11	BioLegend	# 150605
Ly6G	PE	1A8	BioLegend	# 127602
Ly6C	AF700	HK1.4	BioLegend	# 128023
Live dead	Zombie aqua		BioLegend	# 423102

Fibroblast, Myeloid cells, Endothelial cell				
Antibodies	Fluorescein	Clone	Source	Identifier
CD45.2	eF450	104	Thermo Fisher	# 48-0454-82
CD31	APC	390	BioLegend	# 102410
CD11b	APC-Cy7	M1-70	BioLegend	# 101226
TER119	PE	TER 119	Thermo Fisher	# 12-5921-81
PDGFRα	FITC	APA5	Thermo Fisher	# 11-1401-80
Sca-1	PE-Cy7	D7	BioLegend	# 108113
Live dead	Zombie aqua		BioLegend	# 423102
