# Supplementary material for: The Cancer Therapy-Related Clonal Hematopoiesis Driver Gene Ppm1d Promotes Inflammation and Non-Ischemic Heart Failure in Mice
Source: Circ Res. 2021 Jul 28;129(6):684–98. doi: 10.1161/CIRCRESAHA.121.319314 (PMC8409899; doi:10.1161/CIRCRESAHA.121.319314)
Supplement: Supplementary file 3 [file res-129-684-s003.pdf]

Full unedited gel for Fig.4A

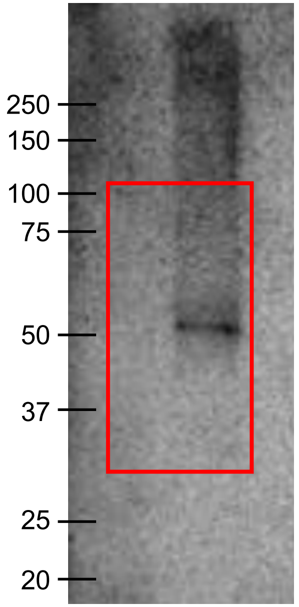

PPM1D

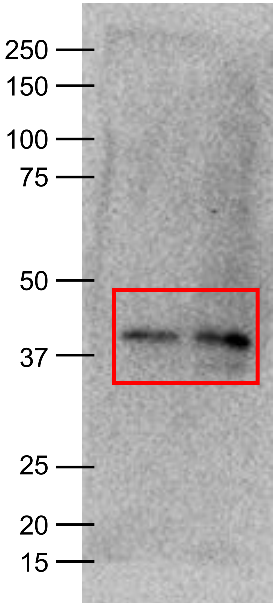

GAPDH

Full unedited gel for Fig.4B

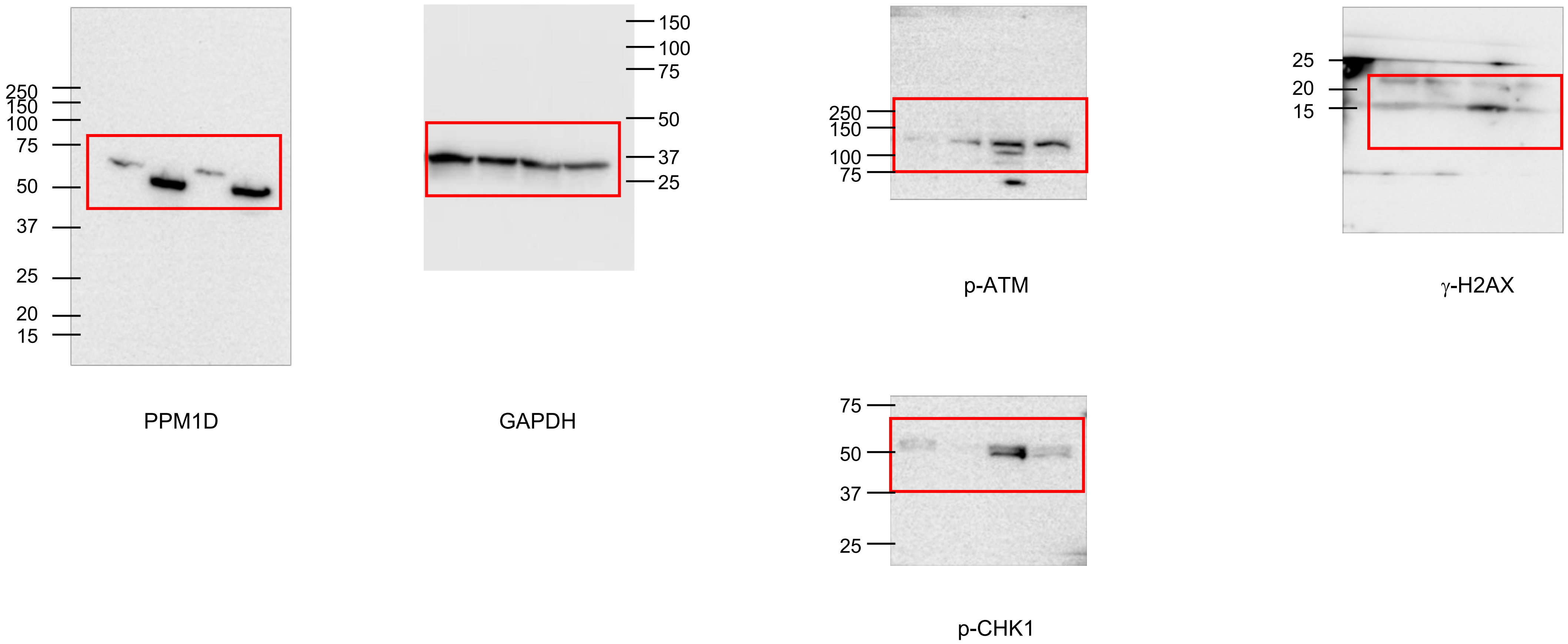

Full unedited gel for Supplemental Fig.VI A

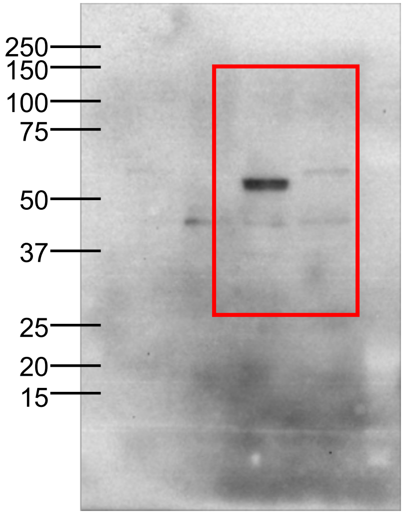

PPM1D

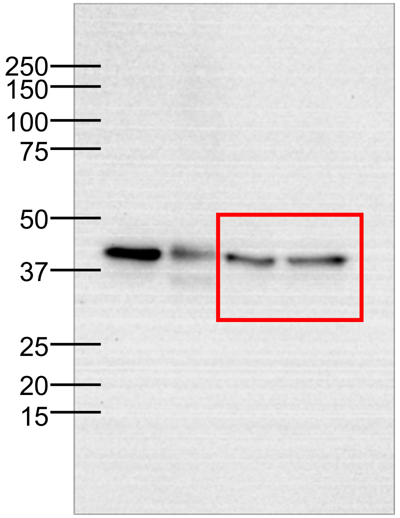

GAPDH

Full unedited gel for Supplemental Fig.XII

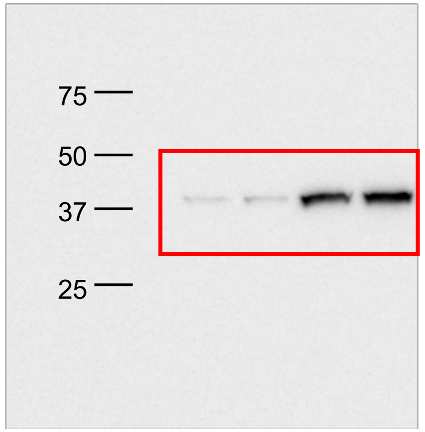

P-p38

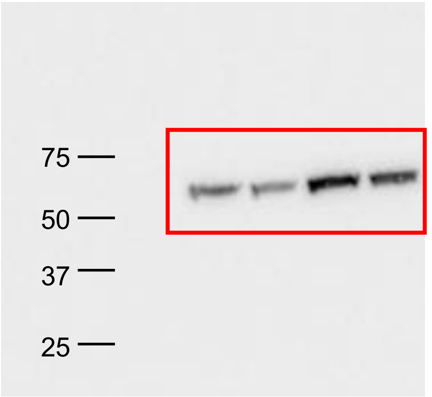

P-p65

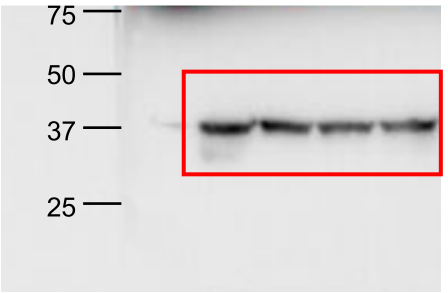

GAPDH

Full unedited gel for Supplemental Fig.XIV

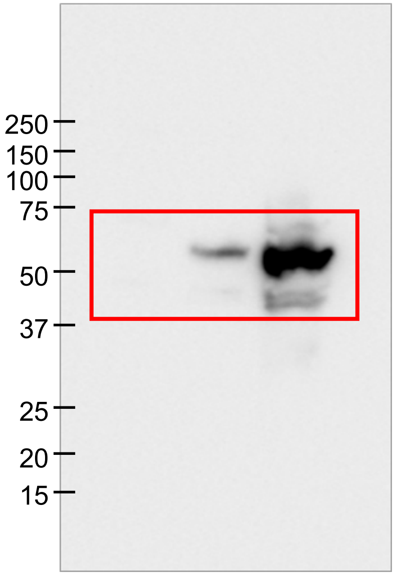

PPM1D

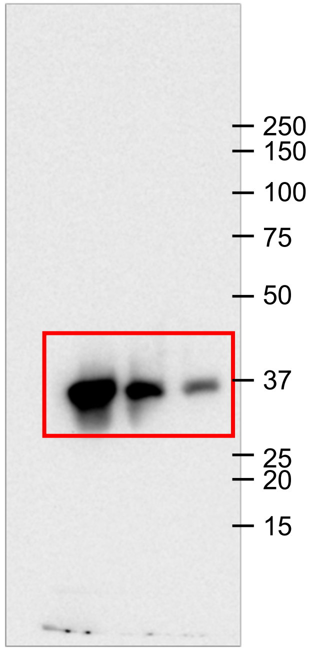

GAPDH
