# Supplementary material for: The Cancer Therapy-Related Clonal Hematopoiesis Driver Gene Ppm1d Promotes Inflammation and Non-Ischemic Heart Failure in Mice
Source: Circ Res. 2021 Jul 28;129(6):684–98. doi: 10.1161/CIRCRESAHA.121.319314 (PMC8409899; doi:10.1161/CIRCRESAHA.121.319314)
Supplement: Supplementary file 4 [file res-129-684-s004.pdf]

Major Resources Table

Animals (in vivo studies)

Species	Vendor or Source	Background Strain	Sex	Persistent ID / URL
C57BL/6J	Jackson Laboratories	C57BL/6J	M	000664
B6(C)-Ccr2tm1.1Cln/J	Jackson Laboratories	C57BL/6J	M	027619
B6(C)-Gt(ROSA)26Sorem1.1(CAG-cas9*,-EGFP)Rsky/J	Jackson Laboratories	C57BL/6J	M	028555

Antibodies

Antibody for Western blotting and Immunostaining

Target antigen	Vendor or Source	Catalog #	Working concentration	Lot # (preferred but not required)	Persistent ID / URL
Phospho-Atm (Ser1981)	Cell Signaling Technology	#13050	1:1000	3	
Phospho-Chk1 (Ser345)	Cell Signaling Technology	#2341	1:1000	8	
Phospho-Histone H2A.X (Ser139)	Cell Signaling Technology	#9718	1:1000	17	
Phospho-p38 (Thr180/Tyr182)	Cell Signaling Technology	#9211	1:1000	9	
Phospho-p65 (Ser536)	Cell Signaling Technology	#3033	1:1000	17	
WIP1	Cell Signaling Technology	#11901	1:1000	1	
GAPDH	Cell Signaling Technology	#2118	1:1000		
IL-1β	Bioss	#BS-6319R	1:200	AI01163782	
Mac-3	Santa Cruz Biotechnology	#sc-19991	1:200	#D2611	
Rabbit IgG-HRP	Santa Cruz Biotechnology	#sc-2357	1:5000	#E1618	
Alexa Flour 594-conjugated anti-rat IgG	Life Technologies	#A-21209	1:1000	#1398009	

Antibodies used for flow cytometry analysis.

Peripheral Blood

Antibodies	Fluorescein	Clone	Source	Identifier
CD115	PE-Cy7	AFS98	Thermo Fisher	# 25-1152-82

DOI [to be added]

Ly6C	APC	AL21	BD Biosciences	# 560595
Ly6G	PerCP5.5	1A8	BD Biosciences	# 560602
B220	APC-Cy7	RA3-6B2	BD Biosciences	# 552094
CD3	BV711	145-2C11	BioLegend	# 100349
CD4	FITC	RM4-5	Thermo Fisher	# 11-0042-82
CD8a	BV510	53.6-7	BioLegend	# 100751

Heart 1

Antibodies	Fluorescein	Clone	Source	Identifier
CD45.2	PerCP5.5	104	BioLegend	# 109828
CD64	BV711	X54-5/7.1	BioLegend	# 139311
CCR2	BV421	SA203G11	BioLegend	# 150605
Ly6G	PE-Cy7	1A8	BioLegend	# 127618
Ly6C	FITC	HK1.4	BioLegend	# 128006
Live dead	Zombie aqua		BioLegend	# 423102

Heart 2

Antibodies	Fluorescein	Clone	Source	Identifier
CD45.2	PerCP5.5	104	BioLegend	# 109828
CD11b	AF700	M1/70	BioLegend	#101222
CD3	PE-eFlour610	145-2C11	Thermo Fisher	# 61-0031-82
CD8a	BV510	1A8	BioLegend	# 100752
CD4	FITC	RM4-5	Thermo Fisher	# 11-0042-82
CD19	APC-Cy7	SJ25C1	BioLegend	# 363009
Live dead	Zombie violet		BioLegend	# 423113

Heart 3

Antibodies	Fluorescein	Clone	Source	Identifier
CD45.2	PerCP5.5	104	BioLegend	# 109828
CD11b	APC-Cy7	M1-70	BioLegend	# 101226
Ly6G	PE	1A8	BioLegend	# 127602

DOI [to be added]

Ly6C	APC	AL-21	BD Biosciences	# 560595
F4/80	PE-Cy7	BM8	BioLegend	# 123116
Live dead	Zombie aqua		BioLegend	# 423102

Heart 4				
Antibodies	Fluorescein	Clone	Source	Identifier
CD45.2	PerCP5.5	104	BioLegend	# 109828
CD64	APC	X54-5/7.1	BioLegend	# 139306
CCR2	BV421	SA203G11	BioLegend	# 150605
Ly6G	PE	1A8	BioLegend	# 127602
Ly6C	AF700	HK1.4	BioLegend	# 128023
Live dead	Zombie aqua		BioLegend	# 423102

Fibroblast, Myeloid cells, Endothelial cell				
Antibodies	Fluorescein	Clone	Source	Identifier
CD45.2	eF450	104	Thermo Fisher	# 48-0454-82
CD31	APC	390	BioLegend	# 102410
CD11b	APC-Cy7	M1-70	BioLegend	# 101226
TER119	PE	TER 119	Thermo Fisher	# 12-5921-81
PDGFRα	FITC	APA5	Thermo Fisher	# 11-1401-80
Sca-1	PE-Cy7	D7	BioLegend	# 108113
Live dead	Zombie aqua		BioLegend	# 423102

DNA/cDNA Clones

Clone Name	Sequence	Source / Repository	Persistent ID / URL
pLKO5.sgRNA.EFS.tRFP		Addgene	#57823
pLKO5.sgRNA.EFS.GFP		Addgene	#57822
LentiCRISPRv2GFP		Addgene	#82416
psPAX2		Addgene	#12260
pMD2.G		Addgene	#12259

Cultured Cells

DOI [to be added]

Name	Vendor or Source	Sex (F, M, or unknown)	Persistent ID / URL
HEK 293T	American Type Culture Collection	F	
J774.1	American Type Culture Collection	unknown	

Data & Code Availability: N/A

Description	Source / Repository	Persistent ID / URL

Other: N/A

Description	Source / Repository	Persistent ID / URL

DOI [to be added]
